# Inverse Analytical Formula for the Correction of Severe Barrel Lens Distortion Modelled by a Depressed Radial Distortion Polynomial

**DOI:** 10.3390/s26061896

**Published:** 2026-03-17

**Authors:** Guy Blanchard Ikokou, Moreblessings Shoko, Naa Dedei Tagoe

**Affiliations:** 1 Geomatics Department, Faculty of Engineering and Build Environment, Pretoria Campus, Tshwane University of Technology, Pretoria 0183, South Africa; 2 Department of Architecture, Planning and Geomatics, University of Cape Town, Cape Town 7700, South Africa; 3 Department of Geomatic and Civil Engineering, School of Railways and Infrastructure Development, University of Mines and Technology (UMaT), Tarkwa P.O. Box 237, Ghana

**Keywords:** radial lens distortion calibration algorithms, radial lens distortion polynomials, optical radial lens distortion modelling, computer vision, lens distortion inverse modelling

## Abstract

Accurate correction of radial lens distortion is a fundamental requirement in computer vision and photogrammetry, as geometric inaccuracies directly affect 3D reconstruction, mapping, and geospatial measurements, particularly in high-precision imaging systems. In this study, we propose a fully analytical, non-iterative method for truncated inverse modeling of radial lens distortion, applicable to general radial distortion polynomials that contain constant terms. Unlike classical truncated Lagrange series reversion, which relies on recursive expansion and combinatorial series construction, the proposed formulation determines inverse distortion coefficients directly through a system of constrained algebraic inverse polynomials. This enables deterministic computation of inverse parameters without iterative refinement, numerical root finding, or combinatorial complexity. The method was evaluated using ultra-wide-angle smartphone camera imagery exhibiting severe barrel distortion modeled by an eighth-degree depressed radial distortion polynomial. Its performance was compared with a commonly used iterative inverse modeling approach. The analytical formulation demonstrated improved numerical stability and substantially reduced reprojection errors when correcting highly nonlinear distortion profiles, achieving sub-pixel accuracy in image rectification. In contrast, the iterative approach exhibited instability and significantly larger reprojection errors under identical conditions. These results demonstrate that the proposed framework provides a general, robust, and repeatable solution for inverse radial distortion modeling, particularly for high-order polynomial models. The method offers clear practical advantages for camera calibration pipelines in photogrammetry, remote sensing, robotics, and other applications requiring high-fidelity imaging.

## 1. Introduction

Lens distortion remains a critical concern in photogrammetric and computer vision applications, where the geometric fidelity of image data directly influences the accuracy of tasks such as 3D reconstruction, camera calibration, and metric measurements. Distortions introduced by imaging optics are conventionally classified into three categories: radial, tangential, and thin-prism distortions [1,2]. While thin-prism distortions are often negligible due to advancements in modern lens manufacturing, radial and tangential distortions continue to pose substantial challenges in achieving high-precision calibration [3,4]. Among these, radial distortions, which manifest as barrel, pincushion, or moustache distortion profiles, are the most prominent, often resulting in significant displacement of image points either toward or away from the image center [5]. Barrel distortion causes outward displacement, while pincushion distortion pulls points inward [6,7]. To mathematically model radial distortions, odd-power polynomial functions have become standard, originating from the work of [8] and extended by [9], with subsequent refinements from researchers including [10,11,12,13]. These models are particularly advantageous for representing both positive and negative distortion profiles over symmetric radial domains, and they support continuous function expansion across the image plane [14]. In parallel, even-power and mixed-power polynomial models, including depressed and truncated forms, have also been investigated to better model complex distortion patterns [14,15]. These models often include a unit constant term that enforces geometric constraints and maintains continuity at the image center [16,17,18].

In practical calibration workflows, the coefficients of the radial distortion polynomial determine the nonlinearity and severity of distortion. Simplified polynomial forms, such as truncated or depressed models, have been proposed to reduce computational complexity [19,20], whereas high-degree polynomials allow for enhanced modelling flexibility, particularly in representing moustache distortions via alternating sign coefficients [21,22]. Despite the descriptive power of these models, inverse modeling of radial distortion, a crucial step in image rectification, remains an open computational challenge, especially for high-degree polynomials with multiple distortion terms [23,24]. Previous work by [25] emphasizes that the absence of closed-form inverse distortion models introduces significant computational complexity in camera calibration. Reference [26] employed empirical sign adjustments in early approaches such as the cubic inverse model, but these approaches were limited to low-degree models and lacked scalability. Modern calibration platforms like MATLAB, Micmac, and Photomodeler typically require up to three distortion coefficients, beyond the scope of early inverse strategies [27]. Iterative methods remain widespread [28,29], yet they are often sensitive to initial estimates, prone to local minima, and computationally expensive. Other techniques, including lookup table methods, Taylor series approximations, and partial derivative-based solutions, offer limited accuracy across wider distortion ranges or introduce constraints on coefficient domains [30,31]. These methods tend to lose reliability when distortions are severe or uneven, making a direct analytical truncated inverse essential for accurate correction. A widely discussed iterative solution by [32] introduced a recursive polynomial reversion technique, enabling recovery of inverse coefficients through symbolic series expansion. However, this method is structurally rigid and becomes numerically unstable for higher-degree polynomials, as the reversion formulation explicitly truncates the series by setting coefficients beyond the fourth degree (i.e., $\kappa_{5}$, $\kappa_{6}$, $\kappa_{7}$) to zero (see Appendix A), thereby limiting its applicability to high-order radial distortion models and potentially introducing cumulative inaccuracies [33]. The method becomes unstable when scaling factors are poorly constrained or convergence behaves unpredictably across different distortion profiles [34,35].

We present a fully analytical, non-iterative method for inverse modeling of radial distortion polynomials. By reformulating the inverse problem as a constrained algebraic inversion framework, the method directly computes inverse distortion coefficients for arbitrary-degree radial polynomials without relying on iterative solvers, lookup tables, or recursive approximations. By eliminating the need for prior assumptions on radial distances or distortion coefficients, this framework delivers enhanced flexibility, numerical stability, and accuracy, even in the presence of severe barrel distortions modeled with high-degree depressed polynomials. To the best of our knowledge, no existing method in the current literature provides a general and explicitly derived analytical inverse solution applicable to high-order radial distortion models of arbitrary complexity. This work therefore introduces a significant advancement in camera calibration techniques and provides a robust computational tool for distortion correction in modern photogrammetric and computer vision systems.

## 2. Materials and Methods

### 2.1. Optical Sensor and Calibration Hardware

To evaluate the effectiveness of the proposed inverse radial distortion polynomial model, experimental testing was conducted using imagery acquired with a Xiaomi Redmi Pro smartphone (Xiaomi Corporation, Beijing, China). This device features an ultra-wide-angle camera lens capable of capturing 3D scenes with an expansive angular field of view (FOV) of 118°. Such a wide FOV inherently introduces significant barrel distortion, particularly toward the periphery of the image frame, conditions that are ideal for rigorously assessing the performance of the developed analytical inversion method. The presence of pronounced distortion in the captured imagery ensures a challenging test environment for validating the stability and accuracy of the computed inverse distortion coefficients. Table 1 summarizes the key specifications of the ultra-wide-angle imaging system used in this study.

### 2.2. Iterative Approach to Inverting Radial Lens Distortion Polynomials

The iterative approach for computing inverse radial distortion coefficients, as introduced by [32], relies on a recursive formulation of the inverse polynomial. In this method, each inverse coefficient is derived sequentially based on the expressions of preceding coefficients, enabling the construction of an approximate inverse function. Following the symbolic derivation, the model applies an iterative numerical refinement procedure to optimize all inverse coefficients simultaneously. The process starts by assuming that the principal point coincides with the image center, which allows an initial estimate of the radial distance. This estimated radial distance is subsequently refined through a nonlinear optimization function, which minimizes the residual distortion across image points. The initial stage of this method, responsible for refining the magnitude of radial distortion, forms the foundation for the subsequent inverse coefficient estimation and is implemented algorithmically as follows.

(1) $$\left\{\begin{array}{c} \mathrm{required}:\;p_{m}\\ p_{m}=p_{n}\\ \mathrm{repeat}\\ d_{r}=\left\|p_{m}\right\|\\ f\left(r\right)=1+\lambda_{1}r^{2}+\lambda_{2}r^{4}+\lambda_{3}r^{6}+\lambda_{4}r^{8}\\ p_{m}=\frac{p_{n}}{f\left(r\right)}\\ \mathrm{until}\;\mathrm{converge}\;\mathrm{of}\;p_{m}\\ \mathrm{return}\;p_{m} \end{array}\right\}$$

Here, the point $p_{m}$ represents a distorted image point and $p_{n}$ its corresponding undistorted (ideal) point, with the Euclidean norm $\left\|p_{m}-p_{n}\right\|$ minimized iteratively until the distortion model converges, as expressed in Equation (1). The radial distance $d_{r}$ is constrained as $d_{r}=\left\|p_{m}\right\|$, indicating the distance from the principal point $p\left(x_{c},y_{c}\right)$, assumed to lie at the image center, to the distorted point $p_{m}$. The normalized radial distance is computed as follows:

(2) $$r=\sqrt{x_{n}^{2}+y_{n}^{2}}$$

The normalized image point coordinates are computed as follows:

(3) $$x_{n}=\frac{x-x_{c}}{f_{x}}$$

(4) $$y_{n}=\frac{y-y_{c}}{f_{y}}$$

wherein $f_{x}$ and $f_{y}$ are the effective focal lengths. Upon analytical determination of the first inverse distortion coefficient, the remaining coefficients are subsequently derived in a recursive manner. Within this framework, the first four inverse coefficients are explicitly obtained for a depressed eighth-degree even-power radial distortion polynomial, enabling an iterative analytical inversion of the distortion model with improved numerical stability and accuracy. The proposed first four inverse radial distortion coefficients of a depressed eight-degree even power polynomial model are given as follows:

(5) $$\omega_{1}=-\lambda_{1}$$

(6) $$\omega_{2}=3\lambda_{1}^{2}-\lambda_{2}$$

(7) $$\omega_{3}=-12\lambda_{1}^{3}+8\lambda_{1}\lambda_{2}-\lambda_{3}$$

(8) $$\omega_{4}=55\lambda_{1}^{4}-55\lambda_{1}^{2}\lambda_{2}+5\lambda_{2}^{2}+10\lambda_{1}\lambda_{3}-\lambda_{4}$$

where the variables $\lambda_{1}$, $\lambda_{2}$, $\lambda_{3}$ and $\lambda_{4}$ denote the original radial distortion coefficients characterizing the forward distortion model. The corresponding inverse radial distortion coefficients, denoted by $\omega_{1}$, $\omega_{2}$, $\omega_{3}$ and $\omega_{4}$ are derived through an iterative recursive formulation that approximates the inverse mapping of the distortion function.

### 2.3. Analytical Inverse Formulation for a Reduced Eighth-Degree Radial Lens Distortion Polynomial

Let $f\left(r_{d}\right)$ denote an invertible radial distortion model represented by an eighth-degree polynomial:

(9) $$f\left(r_{u}\right)=\omega_{4}r_{u}^{8}+\omega_{3}r_{u}^{6}+\omega_{2}r_{u}^{4}+\omega_{1}r_{u}^{2}+\omega_{0}r_{u}+1$$

A reformulated version of the polynomial function presented in (9) is given below:

(10) $$f\left(r_{u}\right)-1=\omega_{4}r_{u}^{8}+\omega_{3}r_{u}^{6}+\omega_{2}r_{u}^{4}+\omega_{1}r_{u}^{2}+\omega_{0}r_{u}$$

By introducing the substitution $f\left(r_{u}\right)-1=r_{d}$, the formulation in (10) yields the following differential equation:

(11) $$r_{d}=\omega_{4}r_{u}^{8}+\omega_{3}r_{u}^{6}+\omega_{2}r_{u}^{4}+\omega_{1}r_{u}^{2}+\omega_{0}r_{u}$$

Since the original distortion model is represented by an eighth-order polynomial, its inverse is also assumed to be an eighth-order polynomial. To obtain the inverse formulation, it is necessary to express it in terms of the undistorted radius $r_{u}$. The inverse distortion function, $g\left(r_{d}\right)$ is therefore defined as an eighth-order polynomial of the following form:

(12) $$g\left(r_{d}\right)=\lambda_{4}r_{d}^{8}+\lambda_{3}r_{d}^{6}+\lambda_{2}r_{d}^{4}+\lambda_{1}r_{d}^{2}+\lambda_{0}r_{d}+1$$

Introducing the relation $g\left(r_{d}\right)-1=r_{u}$ the equation in (12) can be equivalently expressed as the following differential equation:

(13) $$r_{u}=\lambda_{4}r_{d}^{8}+\lambda_{3}r_{d}^{6}+\lambda_{2}r_{d}^{4}+\lambda_{1}r_{d}^{2}+\lambda_{0}r_{d}$$

By normalizing Equation (11) with respect to the coefficient $\omega_{0}$, the expression simplifies to the following form:

(14) $$r_{u}=\frac{r_{d}}{\omega_{0}}-\frac{1}{\omega_{0}}\left(\omega_{4}r_{u}^{8}+\omega_{3}r_{u}^{6}+\omega_{2}r_{u}^{4}+\omega_{1}r_{u}^{2}\right)$$

The formulation below is obtained by substituting the expression on the right-hand side of (13) into the left-hand side of (14):

(15) $$\lambda_{4}r_{d}^{8}+\lambda_{3}r_{d}^{6}+\lambda_{2}r_{d}^{4}+\lambda_{1}r_{d}^{2}+\lambda_{0}r_{d}=\frac{r_{d}}{\omega_{0}}-\frac{1}{\omega_{0}}\left(\omega_{4}r_{u}^{8}+\omega_{3}r_{u}^{6}+\omega_{2}r_{u}^{4}+\omega_{1}r_{u}^{2}\right)$$

Applying a second-power transformation to Equation (13) produces the following quadratic form:

(16) $$r_{u}^{2}=\lambda_{4}^{2}r_{d}^{16}+2\lambda_{3}\lambda_{4}r_{d}^{14}+2\lambda_{2}\lambda_{4}r_{d}^{12}+\lambda_{3}^{2}r_{d}^{12}+2\lambda_{1}\lambda_{4}r_{d}^{10}+2\lambda_{2}\lambda_{3}r_{d}^{10}+2\lambda_{0}\lambda_{4}r_{d}^{9}+2\lambda_{1}\lambda_{3}r_{d}^{8}\;+\lambda_{2}^{2}r_{d}^{8}+2\lambda_{0}\lambda_{3}r_{d}^{7}+2\lambda_{1}\lambda_{2}r_{d}^{6}+2\lambda_{0}\lambda_{2}r_{d}^{5}+\lambda_{1}^{2}r_{d}^{4}+2\lambda_{0}\lambda_{1}r_{d}^{3}+\lambda_{0}^{2}r_{d}^{2}$$

By discarding all terms of degree higher than eight in Equation (16), the expression simplifies to the following form:

(17) $$r_{u}^{2}=2\lambda_{1}\lambda_{3}r_{d}^{8}+\lambda_{2}^{2}r_{d}^{8}+2\lambda_{0}\lambda_{3}r_{d}^{7}+2\lambda_{1}\lambda_{2}r_{d}^{6}+2\lambda_{0}\lambda_{2}r_{d}^{5}+\lambda_{1}^{2}r_{d}^{4}+2\lambda_{0}\lambda_{1}r_{d}^{3}+\lambda_{0}^{2}r_{d}^{2}$$

Raising Equation (13) to the fourth power and applying algebraic simplifications yields the following expression:

(18) $$r_{u}^{4}=12\lambda_{0}^{2}\lambda_{1}\lambda_{2}r_{d}^{8}+\lambda_{1}^{4}r_{d}^{8}+4\lambda_{0}^{3}\lambda_{2}r_{d}^{7}+4\lambda_{0}\lambda_{1}^{3}r_{d}^{7}+6\lambda_{0}^{2}\lambda_{1}^{2}r_{d}^{6}+4\lambda_{0}^{3}\lambda_{1}r_{d}^{5}+\lambda_{0}^{4}r_{d}^{4}$$

The sixth-power expansion of Equation (13), followed by simplification, results in the following expression:

(19) $$r_{u}^{6}=15\lambda_{0}^{4}\lambda_{1}^{2}r_{d}^{8}+6\lambda_{0}^{5}\lambda_{1}r_{d}^{7}+\lambda_{0}^{6}r_{d}^{6}$$

Applying an eighth-degree transformation to Equation (13) and simplifying the expanded terms, the expression reduces to the following form:

(20) $$r_{u}^{8}=\lambda_{0}^{8}r_{d}^{8}$$

Substituting Equations (17)–(20) into the right-hand side of Equation (15), followed by algebraic rearrangement, yields the following expression:

(21) $$\omega_{4}r_{d}^{8}+\omega_{3}r_{d}^{6}+\omega_{2}r_{d}^{4}+\omega_{1}r_{d}^{2}+\omega_{0}r_{d}=\frac{r_{d}}{\lambda_{0}}-\frac{1}{\lambda_{0}}\left(\begin{array}{l} \left(\begin{array}{l} \omega_{0}^{8}\lambda_{4}+15\omega_{0}^{4}\omega_{1}^{2}\lambda_{3}+12\lambda_{2}\omega_{0}^{2}\omega_{1}\omega_{2}+\lambda_{2}\omega_{1}^{4}\\ +2\lambda_{1}\omega_{1}\omega_{3}+\lambda_{1}\omega_{2}^{2} \end{array}\right)r_{d}^{8}\\ +\left(6\omega_{0}^{5}\omega_{1}\lambda_{3}+2\lambda_{1}\omega_{0}\omega_{3}+4\lambda_{2}\omega_{0}^{3}\omega_{2}+4\omega_{0}\lambda_{2}\omega_{1}^{3}\right)r_{d}^{7}\\ +\left(\omega_{0}^{6}\lambda_{3}+6\omega_{0}^{2}\lambda_{2}\omega_{1}^{2}+2\lambda_{1}\omega_{1}\omega_{2}\right)r_{d}^{6}\\ +\left(4\omega_{0}^{3}\lambda_{2}\omega_{1}+2\lambda_{1}\omega_{0}\omega_{2}\right)r_{d}^{5}\\ +\left(\omega_{0}^{4}\lambda_{2}+\lambda_{1}\omega_{1}^{2}\right)r_{d}^{4}+2\omega_{0}\lambda_{1}\omega_{1}r_{d}^{3}+\omega_{0}^{2}\lambda_{1}r_{d}^{2} \end{array}\right)$$

Equations (11) and (13) define radial distortion polynomials provided the following constraints are fulfilled:

(22) $$\omega_{0}=\lambda_{0}=1$$

Substituting the constraint from (22) into Equation (21) yields the following simplified form:

(23) $$\omega_{4}r_{d}^{8}+\omega_{3}r_{d}^{6}+\omega_{2}r_{d}^{4}+\omega_{1}r_{d}^{2}+r_{d}=r_{d}+\left(\begin{array}{l} \left(\begin{array}{l} -\lambda_{4}-15\omega_{1}^{2}\lambda_{3}-12\lambda_{2}\omega_{1}\omega_{2}-\lambda_{2}\omega_{1}^{4}\\ -2\lambda_{1}\omega_{1}\omega_{3}-\lambda_{1}\omega_{2}^{2} \end{array}\right)r_{d}^{8}\\ +\left(-6\omega_{1}\lambda_{3}-2\lambda_{1}\omega_{3}-4\lambda_{2}\omega_{2}-4\lambda_{2}\omega_{1}^{3}\right)r_{d}^{7}\\ +\left(-\lambda_{3}-6\lambda_{2}\omega_{1}^{2}-2\lambda_{1}\omega_{1}\omega_{2}\right)r_{d}^{6}\\ +\left(-4\lambda_{2}\omega_{1}-2\lambda_{1}\omega_{2}\right)r_{d}^{5}\\ +\left(-\lambda_{2}-\lambda_{1}\omega_{1}^{2}\right)r_{d}^{4}-2\lambda_{1}\omega_{1}r_{d}^{3}-\lambda_{1}r_{d}^{2} \end{array}\right)$$

From the formulation presented in Equation (23), we arrive at the following set of equations.

(24) $$\omega_{1}r_{d}^{2}=-\lambda_{1}r_{d}^{2}$$

(25) $$-2\lambda_{1}\omega_{1}r_{d}^{3}=0$$

(26) $$\omega_{2}r_{d}^{4}=\left(-\lambda_{2}-\lambda_{1}\omega_{1}^{2}\right)r_{d}^{4}$$

(27) $$\left(-4\lambda_{2}\omega_{1}-2\lambda_{1}\omega_{2}\right)r_{d}^{5}=0$$

(28) $$\omega_{3}r_{d}^{6}=\left(-\lambda_{3}-6\lambda_{2}\omega_{1}^{2}-2\lambda_{1}\omega_{1}\omega_{2}\right)r_{d}^{6}$$

(29) $$\left(-6\omega_{1}\lambda_{3}-2\lambda_{1}\omega_{3}-4\lambda_{2}\omega_{2}-4\lambda_{2}\omega_{1}^{3}\right)r_{d}^{7}=0$$

(30) $$\omega_{4}r_{d}^{8}=\left(-\lambda_{4}-15\omega_{1}^{2}\lambda_{3}-12\lambda_{2}\omega_{1}\omega_{2}-\lambda_{2}\omega_{1}^{4}-2\lambda_{1}\omega_{1}\omega_{3}-\lambda_{1}\omega_{2}^{2}\right)r_{d}^{8}$$

Since the depressed polynomial model on the left-hand side of the equality sign in (23) does not contain third-, fifth-, or seventh-degree terms, the corresponding constraints in Equations (25), (27), and (29) are not required for further analysis. Consequently, the relevant relationships are obtained from Equations (24), (26), (28), and (30) as follows:

(31) $$\omega_{1}=-\lambda_{1}$$

Substituting Equation (31) into Equation (26) yields the following expression for the second inverse coefficient:

(32) $$\omega_{2}=-\lambda_{1}^{3}-\lambda_{2}$$

Substituting Equations (31) and (32) into (26) yields the following expression for the third inverse distortion coefficient:

(33) $$\omega_{3}=-2\lambda_{1}^{5}-8\lambda_{1}^{2}\lambda_{2}-\lambda_{3}$$

Substituting Equations (31)–(33) into Equation (30) yields the following expression for the fourth inverse radial distortion coefficient:

(34) $$\omega_{4}=-5\lambda_{1}^{7}-31\lambda_{1}^{4}\lambda_{2}-17\lambda_{1}^{2}\lambda_{3}-13\lambda_{1}\lambda_{2}^{2}-\lambda_{4}$$

A comparative summary of the inverse distortion coefficient formulations presented in Equations (5)–(8) and (31)–(34) is provided in Table 2. Analysis of the second and third columns reveals that both the iterative and fully analytical calibration methods yield identical expressions for the first inverse radial distortion coefficient $\omega_{1}$. The primary distinction between the two methods emerges in the formulation of the second inverse distortion coefficient $\omega_{2}$. Although both approaches incorporate a common second term involving the second forward distortion coefficient $\lambda_{2}$, they differ significantly in the structure of the first term. The iterative method features a positive first term with a numerical multiplier of 3 and a quadratic dependence on the first forward distortion coefficient $\lambda_{1}$. In contrast, the fully analytical formulation yields a negative first term with a numerical coefficient of 1 and a cubic dependence on $\lambda_{1}$. Regarding the third inverse distortion coefficient $\omega_{3}$, both approaches produce expressions with the same number of terms and share a common structure in the first term, which is composed solely of the coefficient $\lambda_{1}$ and carries a negative sign. However, the magnitude of the associated numerical coefficient differs: the iterative method results in a larger (more negative) coefficient, while the fully analytical formulation leads to a smaller negative coefficient. This distinction reflects differences in the derivation strategies, particularly in how the contributions of higher-order distortion terms are handled.

Further examination of the third inverse radial distortion coefficient $\omega_{3}$ reveals additional structural similarities and distinctions between the iterative and fully analytical calibration approaches. Both formulations share a common third term composed solely of the third radial distortion coefficient $\lambda_{3}$. However, differences arise in the numerical coefficients and the exponentiation of variables within the respective terms. In both methods, the second term consists of a product involving $\lambda_{1}$ and $\lambda_{2}$; yet, in the fully analytical approach, $\lambda_{1}$ is raised to the second power, whereas in the iterative formulation, $\lambda_{1}$ appears with a linear exponent. Additionally, this term is scaled by a positive numerical coefficient of 8 in the iterative approach, in contrast to a negative coefficient of equal magnitude in the fully analytical counterpart. Turning to the fourth inverse radial distortion coefficient $\omega_{4}$, both calibration strategies yield expressions with the same number of terms and share a common fifth term, which is comprised solely of the fourth radial distortion coefficient $\lambda_{4}$. While the first four terms in both methods are constructed from the same sequence of original distortion coefficients, their formulations differ in terms of the powers applied to these coefficients and the magnitudes and signs of their respective numerical multipliers. The first term in the iterative formulation involves $\lambda_{1}$ raised to the fourth power and is scaled by a relatively large positive coefficient. Conversely, in the fully analytical approach, this term involves raising $\lambda_{1}$ to the seventh power and is associated with a smaller negative coefficient. The second term comprises the product $\lambda_{1}\lambda_{3}$; in the iterative strategy, both coefficients are of linear order, while in the fully analytical method, the coefficient $\lambda_{1}$ is squared. Furthermore, the associated numerical coefficient is smaller and positive in the iterative approach, and larger but negative in the fully analytical counterpart. The third term is formed by the product of $\lambda_{1}$ and $\lambda_{2}$. In the iterative method, $\lambda_{1}$ is raised to the second power, while in the fully analytical case, it is raised to the fourth power. The iterative formulation assigns this term a larger negative coefficient, whereas the fully analytical version uses a smaller negative coefficient. Notably, the fourth term differs in structure between the two methods. In the iterative calibration, it consists solely of $\lambda_{2}$ squared, while in the fully analytical formulation, it is composed of the product $\lambda_{1}\lambda_{2}^{2}$. The iterative approach associates this term with a small positive numerical coefficient, while the fully analytical method applies a larger negative coefficient. Despite these differences, both calibration strategies converge on the same fifth term, which is simply the fourth distortion coefficient $\lambda_{4}$, underscoring some consistency in the treatment of higher-order terms.

### 2.4. Calibration-Based Estimation of Radial Distortion Coefficients

In this study, MATLAB R2018b (Version R2018b, MathWorks, Natick, MA, USA) was utilized for camera calibration, leveraging its built-in calibration toolbox that supports both the standard pinhole model and the fisheye distortion model. The fisheye model does not estimate radial distortion coefficients explicitly, whereas the standard model allows for the inclusion of up to three radial distortion parameters. To ensure accurate spatial scaling during calibration, the checkerboard used featured a 7 × 5 square pattern, with each black square precisely measuring 30 mm per side. The calibration process focused exclusively on radial distortion, and therefore, tangential distortion coefficients and the skew parameter were excluded from estimation. Additionally, no initial values were provided for the radial distortion coefficients during the nonlinear optimization stage, due to the challenges associated with selecting suitable initial estimates without prior knowledge of the lens characteristics. Image acquisition was performed using a Xiaomi Redmi Note 11 Pro smartphone, capturing 40 images of the calibration checkerboard affixed to a planar whiteboard mounted against a flat wall. The MATLAB toolbox supports calibration with either connected webcams or externally captured imagery. To ensure robust and consistent corner detection, the selected checkerboard pattern intentionally avoided a square grid layout (e.g., 7 × 7 or 8 × 8) to prevent ambiguity in origin identification across the image set. Maintaining the same corner as the origin of the 3D coordinate system in all images is critical, and non-square patterns assist the algorithm in reliably detecting this reference point. Following image capture, all 40 images were imported into the MATLAB workspace, and corner detection was executed using the toolbox utilities. Five images were rejected during this stage. Upon closer examination, we found that the rejected images were compromised by unfavorable imaging conditions. In some cases, direct overhead lighting caused overexposure, whereas in others, extreme oblique viewing angles reduced the visibility and detectability of distant corners. Finally, during the calibration setup, the 30 mm square size was explicitly defined within the toolbox interface. Accurate specification of this dimension is essential, as it directly influences the computation of 3D–2D point correspondences and thus the reliability of the intrinsic and distortion parameter estimates. The computed radial distortion coefficients, listed in Table 3, represent the lens distortion characteristics estimated from the calibration image set using MATLAB’s camera calibration toolbox.

### 2.5. Inverse Radial Distortion Modelling for Severe Barrel Deformation

Based on the iterative inverse coefficients presented in Equations (5)–(8), in conjunction with the estimated radial distortion coefficients summarized in Table 3, the inverse radial distortion models can be systematically formulated to compensate for the radial distortions introduced by the Xiaomi Redmi Pro 11 lens system. These distortions are characterized by a depressed eighth-degree polynomial function, and the proposed inverse models provide an effective means of geometric correction within the imaging pipeline. The inverse radial distortion models, derived to correct lens-induced distortions characterized by a depressed eighth-degree polynomial, are formulated to compensate for the specific radial distortions introduced by the Xiaomi Redmi Pro 11 camera system, as presented in Equations (35) and (36).

(35) $$x_{u}=x_{d}+x_{d}\left(\begin{array}{c} -\lambda_{1}r_{d}^{2}-\left(3\lambda_{1}^{2}-\lambda_{2}\right)r_{d}^{4}-\left(8\lambda_{1}\lambda_{2}-12\lambda_{1}^{3}-\lambda_{3}\right)r_{d}^{6}\\ -\left(55\lambda_{1}^{4}+10\lambda_{1}\lambda_{3}-55\lambda_{1}^{2}\lambda_{2}+5\lambda_{2}^{2}-\lambda_{4}\right)r_{d}^{8} \end{array}\right)$$

(36) $$y_{u}=y_{d}+y_{d}\left(\begin{array}{c} -\lambda_{1}r_{d}^{2}-\left(3\lambda_{1}^{2}-\lambda_{2}\right)r_{d}^{4}-\left(8\lambda_{1}\lambda_{2}-12\lambda_{1}^{3}-\lambda_{3}\right)r_{d}^{6}\\ -\left(55\lambda_{1}^{4}+10\lambda_{1}\lambda_{3}-55\lambda_{1}^{2}\lambda_{2}+5\lambda_{2}^{2}-\lambda_{4}\right)r_{d}^{8} \end{array}\right)$$

The radial distortions introduced by the Xiaomi Redmi Note 11 lens system, characterized by an eighth-order depressed radial distortion polynomial, can be effectively corrected using analytically derived inverse distortion coefficients. This correction is achieved through a set of inverse transformation equations formulated as follows:

(37) $$x_{u}=x_{d}+x_{d}\left(\begin{array}{c} -\lambda_{1}r_{d}^{2}-\left(\lambda_{1}^{3}+\lambda_{2}\right)r_{d}^{4}-\left(2\lambda_{1}^{5}+8\lambda_{1}^{2}\lambda_{2}+\lambda_{3}\right)r_{d}^{6}\\ -\left(5\lambda_{1}^{7}+31\lambda_{1}^{4}\lambda_{2}+17\lambda_{1}^{2}\lambda_{3}+13\lambda_{1}\lambda_{2}^{2}+\lambda_{4}\right)r_{d}^{8} \end{array}\right)$$

(38) $$y_{u}=y_{d}+y_{d}\left(\begin{array}{c} -\lambda_{1}r_{d}^{2}-\left(\lambda_{1}^{3}+\lambda_{2}\right)r_{d}^{4}-\left(2\lambda_{1}^{5}+8\lambda_{1}^{2}\lambda_{2}+\lambda_{3}\right)r_{d}^{6}\\ -\left(5\lambda_{1}^{7}+31\lambda_{1}^{4}\lambda_{2}+17\lambda_{1}^{2}\lambda_{3}+13\lambda_{1}\lambda_{2}^{2}+\lambda_{4}\right)r_{d}^{8} \end{array}\right)$$

The observed variation in the numerical accuracy of the estimated inverse coefficients, particularly for higher-order terms, can be attributed to their increased sensitivity to measurement noise and truncation effects inherent in finite-order polynomial expansions. Lower-order coefficients, such as $\omega_{1}$ and $\omega_{2}$, predominantly govern distortion behaviour in the central region of the field of view and therefore tend to be more stable under small perturbations in the forward distortion parameters. In contrast, higher-order coefficients, such as $\omega_{3}$ and $\omega_{4}$, are more strongly influenced by peripheral image observations, where distortion magnitudes are larger and measurement uncertainties are amplified. Moreover, the higher-degree algebraic combinations of $\lambda_{1}$, $\lambda_{2}$, $\lambda_{3}$ and $\lambda_{4}$ introduce compounded numerical effects, which further increase sensitivity to small estimation errors. This behaviour explains the varying levels of accuracy reported in Table 3 and highlights the importance of numerical stability considerations when deriving and implementing high-order inverse radial distortion models.

## 3. Results

### 3.1. Inverse Modelling Assessment

The radial distortion coefficients estimated using the MATLAB camera calibration toolbox, as reported in Table 3, exhibit alternating signs. While the occurrence of coefficients with differing signs has been associated in the literature with complex distortion profiles such as moustache distortion, this condition alone is not a definitive indicator of its presence. To reduce ambiguity and facilitate more meaningful interpretation of the coefficients $\lambda_{1}$, $\lambda_{2}$ and $\lambda_{3}$, we applied a rescaling procedure. Specifically, $\lambda_{2}$ was normalized by a factor of $10^{1}$, and $\lambda_{3}$ by $10^{1}$, effectively normalizing all values to the $10^{-1}$ range. A rescaling of the coefficients presented in Table 3 enhances comparability and facilitates analysis of their relative magnitudes and influence on the distortion profile.

Given its relatively large numerical magnitude, the leading radial distortion coefficient $\lambda_{1}$ is anticipated to exert the most significant influence on the overall distortion function comprising all four estimated parameters. Substituting the rescaled coefficient estimates into Equations (35)–(36) and (37)–(38) facilitates a direct comparative analysis of the inverse radial distortion models derived from the two calibration strategies. Table 4 summarizes the estimated inverse radial distortion coefficients for each method, thereby facilitating a quantitative evaluation of their respective distortion compensation behaviours.

**Figure 1:**
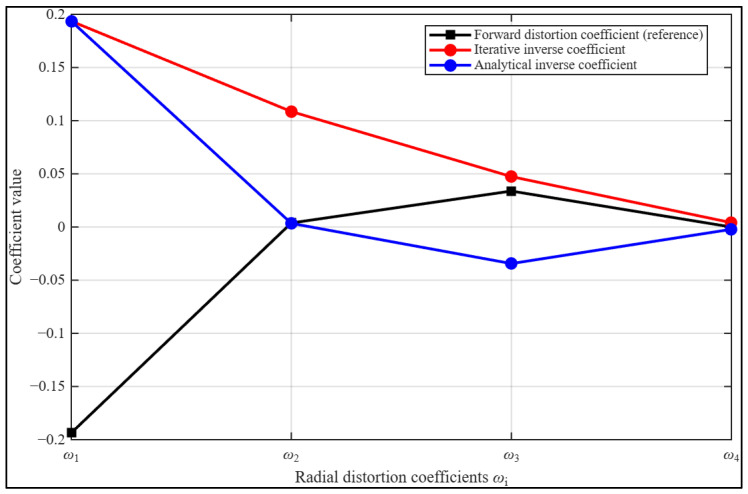
Comparison between the inverse radial distortion curves derived from the analytical and iterative calibration strategies relative to the original distortion function.

Figure 1 presents the estimated inverse radial distortion curves obtained from two calibration approaches. The red curve corresponds to the iterative calibration strategy [33] applied to model the inverse of an eight-degree radial distortion polynomial. The grey curve represents the analytically derived inverse function addressing the same eight-degree radial distortion.

To enable a clear visual comparison, the inverse coefficients for both approaches were scaled uniformly. Specifically, the first inverse coefficient was multiplied by 10^3^, the second by 10^7^, and the third by 10^11^. This scaling aligns the curves optimally, allowing structural differences between the two methods to be observed directly. The iterative curve highlights the contributions of lower-order distortion terms more prominently, while the analytically derived curve emphasizes higher-order effects, reflecting the distinct derivation strategies of the two approaches.

Between the second and third coefficients, the behaviour of the two inversion strategies diverges. The iterative curve remains positive over a broader range and follows a smooth convex parabolic trajectory before gradually approaching zero at the fourth coefficient. By comparison, the analytical curve decreases more quickly and crosses below zero soon after the second coefficient, highlighting its greater sensitivity to the higher-order components of the polynomial.

Although ideal radial distortion is typically monotonic within the usable field of view, polynomial inverse approximations, particularly for high-degree models, may exhibit localized non-monotonic characteristics depending on truncation effects and coefficient estimation stability. Notably, the analytical inverse function demonstrates near-symmetry with respect to the original distortion curve about the horizontal axis, suggesting a closer functional inversion of the distortion profile.

These observations indicate that the analytical formulation more faithfully captures the higher-order structure of the eight-degree depressed polynomial distortion model, thereby providing improved correction performance while preserving global monotonic consistency within the operational image domain.

### 3.2. Model-Based Correction of Severe Nonlinear Barrel Distortions Characterized by an Eighth-Degree Polynomial

The severe barrel distortions were corrected using both iterative models (Equations (35) and (36)) and analytically derived models (Equations (37) and (38)). Corrections were applied to 54 distorted image points measured from a checkerboard calibration pattern captured with a Xiaomi Redmi 11 Pro camera. The reference distortion-free image coordinates were obtained using the MATLAB Camera Calibration Toolbox. During calibration, the intrinsic parameters and lens distortion coefficients were first estimated from multiple images of a planar calibration pattern. Using these parameters, the detected image points were corrected for lens distortion to obtain their undistorted (ideal) image coordinates. The planar calibration grid points $\left(Z=0\right)$ were then mapped to the image plane via the estimated homography matrix. In MATLAB, this transformation was implemented as a projective geometric mapping, and the resulting homogeneous coordinates were normalized to obtain the corresponding Cartesian image coordinates. The resulting reference undistorted coordinates are independent of the proposed inverse distortion model and were used solely for validation purposes. Table 5 summarizes the results, showing the corrected coordinates obtained from each method in the last four columns. This presentation highlights the effectiveness of both strategies in compensating for high-order radial distortions, allowing a direct comparison of the iterative and analytical approaches.

Table 5 presents a representative subset of distortion-free reference coordinates together with the corresponding corrected coordinates obtained using the iterative and fully analytical inverse models across selected distortion radii. A direct comparison shows that the iterative model produces larger deviations from the reference coordinates, with both overestimation and underestimation observed across different points. For example, in the first row, the iterative correction shifts the point from (4.70, 3.53) to (5.30, 4.01), indicating noticeable overcorrection. Similar deviations are evident in points located farther from the image center, where distortion effects are more pronounced (e.g., 7.02, 1.51). In contrast, the analytically derived inverse model consistently yields corrected coordinates that remain closer to the distortion-free references. The residual differences are comparatively smaller and more uniformly distributed across the dataset, indicating improved stability and higher inversion fidelity. These results suggest that while the iterative model partially compensates for radial distortion, it exhibits greater sensitivity to higher-order effects, leading to amplified residual errors, particularly toward the image periphery. The fully analytical formulation, by contrast, demonstrates stronger numerical consistency and more accurate reconstruction of the undistorted coordinate positions.

**Figure 2:**
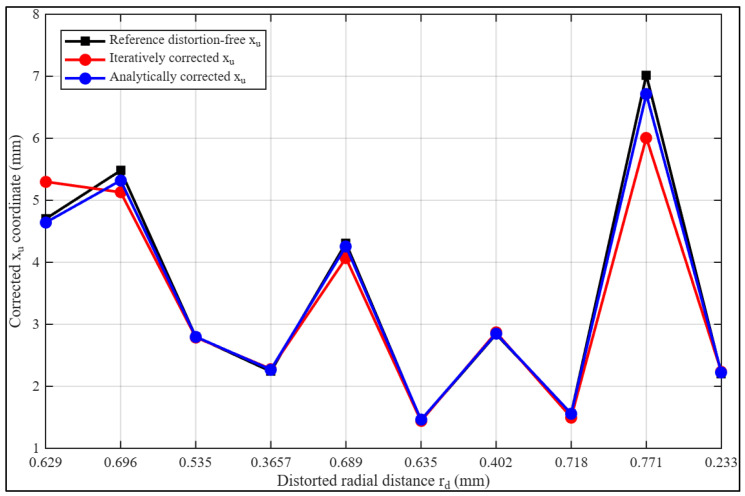
Severe barrel radial distortion correction curves per calibration method for the correction of image $x_{d}$ coordinates.

To enhance interpretability beyond the numerical comparison, the coordinate sets are visualized in Figure 2. The analytical curve closely overlaps the reference trajectory, indicating strong geometric agreement with the distortion-free coordinates. In contrast, the iterative curve progressively deviates from the reference and exhibits noticeable residual distortion, particularly toward regions of stronger radial displacement.

The close correspondence between the analytically corrected coordinates and the distortion-free references in Table 5 confirms that the inverse coefficients derived in Equations (31)–(34) more effectively compensate for the severe barrel distortion introduced by coefficients $\kappa_{1}$, $\kappa_{2}$ and $\kappa_{3}$. By comparison, the iterative formulation in Equations (5)–(7) demonstrates larger residual deviations, reflecting reduced accuracy in modelling higher-order distortion effects.

Together, the tabulated results and their graphical representation in Figure 2 provide consistent quantitative and qualitative evidence of the improved stability and correction accuracy achieved by the fully analytical inversion approach across the dataset. The analytical curve (blue) nearly overlaps the reference distortion-free curve, closely following the trajectory of the ideal undistorted coordinates. In contrast, the iterative curve (red) deviates from the reference, intersecting the other curves at several points and indicating residual distortion. These observations demonstrate the improved performance of the analytical inversion approach in compensating for high-order radial distortion, resulting in more reliable correction across the dataset.

The agreement between the distortion-free image coordinates estimated using the two correction approaches and their corresponding reference values is illustrated by the results summarized in Table 5 and Figure 2. While these indicators confirm the overall accuracy of the correction strategies, they do not explicitly reveal how the corrected coordinates are positioned relative to the original distorted measurements. This spatial relationship is critical for assessing whether a correction method produces physically consistent point displacements rather than merely minimizing residual error. To address this limitation, Table 6 presents the distribution of a subset of corrected $x_{u}$ coordinates relative to the interval defined by the measured distorted coordinates $x_{d}$ and their corresponding distortion-free references. Ideally, the corrected values should lie within this bounded interval, reflecting proper inversion of the distortion effect.

Examination of the results shows that the analytically derived inverse model consistently produces corrected coordinates that fall within the expected bounds and remain close to the reference distortion-free values. The deviations are comparatively small and uniformly distributed across the dataset.

In contrast, the iterative method exhibits larger departures from the reference values. In several cases (e.g., 4.70 → 5.30 and 7.02 → 6.01), the corrected coordinates shift substantially relative to both the distorted and reference measurements, indicating overcorrection or amplified residual error. These deviations are more pronounced for points located farther from the image center, where barrel distortion effects are strongest.

**Figure 3:**
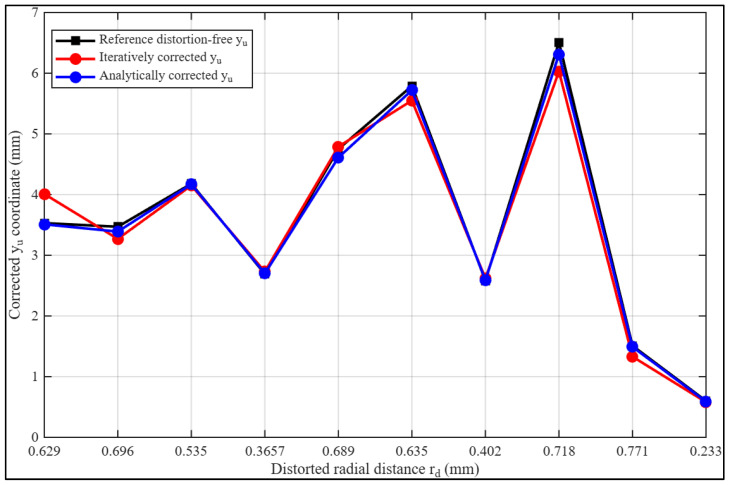
Severe radial distortion correction curves per calibration method for the correction of image $y_{d}$ coordinates.

Figure 3 presents a graphical comparison of the corrected coordinate sets with the distortion-free reference data as a function of radial distortion radius. The analytically corrected curve closely follows the reference trajectory across the image domain, indicating strong geometric consistency. In contrast, the iterative curve exhibits larger deviations, particularly in regions of pronounced radial displacement where barrel distortion effects are strongest.

Consistent with the interval analysis in Table 6, the iterative correction shows greater departures from the bounded range defined by the distorted and reference measurements, reflecting amplified residual error in high-distortion regions. The analytically derived inverse coefficients, however, maintain closer agreement with the reference coordinates across both central and peripheral areas.

Together, the numerical and graphical results demonstrate that the fully analytical inversion model more effectively compensates for the severe barrel distortion introduced by the Xiaomi Redmi 11 Pro lens system, providing improved robustness and higher inversion fidelity under strong distortion conditions.

To assess the positional reliability of the corrected $y_{u}$ image coordinates, the results summarized in Table 7 were evaluated against the intervals defined by the corresponding distorted measurements and their undistorted reference values. The analysis indicates that corrections obtained using the iterative approach remain largely outside the prescribed bounds. Furthermore, comparison with the reference data reveals a systematic tendency for a substantial proportion of the iteratively corrected points to deviate either below or above their corresponding undistorted reference values.

A contrasting behaviour is observed for the coordinates produced using analytically derived inverse coefficients. These corrected points exhibit only marginal departures from the distortion-free references, with deviations that are small and spatially uniform across the image domain. Occasional boundary crossings are limited in magnitude and do not indicate systematic bias. Overall, the boundary-based evaluation reveals a clear performance difference between the two correction strategies. While the iterative formulation respects the geometric constraints of the distorted data, the analytical inverse model more accurately recovers the “true” undistorted $y_{u}$ coordinates. This confirms the superior accuracy and stability of the analytical approach when applied to distortion correction.

### 3.3. Accuracy Assessment

Reprojection accuracy was evaluated using the error formulation defined in Equation (39), and the resulting values for each calibration strategy are summarized in Table 8.

(39) $$\mathrm{reprojection}\;\mathrm{error}=\gamma_{\mathrm{reference}}-\gamma_{\mathrm{computed}}$$

where the parameter $\gamma_{\mathrm{reference}}$ represents the measured reference distortion-free image coordinates of an image point, while $\gamma_{\mathrm{computed}}$ represents the corresponding computed distortion-free image coordinates of the same point.

The error patterns associated with the iterative inverse distortion models reveal a pronounced imbalance in the correction process: although these models aim to compensate for strong barrel distortion, they simultaneously induce noticeable pincushion effects. The comparatively large reprojection errors for both horizontal and vertical image coordinates reflect this behavior. In particular, the mean reprojection error associated with the iterative correction of the $x_{d}$ coordinates is approximately 0.089 mm (0.34 pixels), whereas the vertical component exhibits larger discrepancies, with peak error of about 0.48 mm (1.81 pixels) and an average magnitude of approximately 0.28 mm (1.06 pixels). Such error levels are considered excessive for high-precision photogrammetric and computer-vision applications.

By contrast, the reprojection errors obtained using analytically derived inverse distortion models are consistently small and spatially stable. Average errors of approximately 0.055 mm (0.21 pixels) for the $x_{d}$ component and 0.053 mm (0.20 pixels) for the $y_{d}$ component demonstrate a substantial improvement in correction fidelity. These results indicate that the analytical inverse formulation is far more effective at suppressing the effects of severe barrel radial distortion without introducing secondary geometric artifacts.

Table 9 presents the coordinate-wise reprojection errors for both iterative and analytical inversion methods over a representative set of distorted radii.

The results indicate that the analytical method consistently reduces the magnitude of reprojection errors in both $x_{u}$ and $y_{u}$ compared to the iterative approach. For example, the iterative solution exhibits peak errors of 1.01 mm in $x_{u}$ and 0.48 mm in $y_{u}$, whereas the analytical formulation limits these maximum deviations to 0.31 mm and 0.20 mm, respectively. Several data points show near-zero errors in the analytical solution, reflecting its ability to accurately reconstruct undistorted coordinates across the radial domain. Although certain distorted radii around 0.69 mm exhibit relatively small iterative reprojection errors, this localized behavior does not imply uniform numerical stability across the radial domain. In polynomial radial distortion models, the distortion magnitude increases nonlinearly with radius due to the growing influence of higher-order terms. Within moderate radial intervals, these higher-order contributions remain comparatively small, resulting in reduced curvature of the distortion function and limited propagation of truncation effects in the iterative inversion. However, as the distorted radius exceeds approximately 0.7 mm, higher-order polynomial components become increasingly dominant, intensifying nonlinearity and amplifying approximation errors. This effect explains the substantial residual growth observed at radii such as 0.718 mm and 0.771 mm. In this region, the iterative errors display higher variability and occasional sign oscillations, suggesting sensitivity to truncation or approximation errors.

In contrast, the analytical inversion maintains consistently bounded reprojection errors across the full radial range, demonstrating improved numerical robustness under strongly nonlinear distortion conditions.

**Figure 4:**
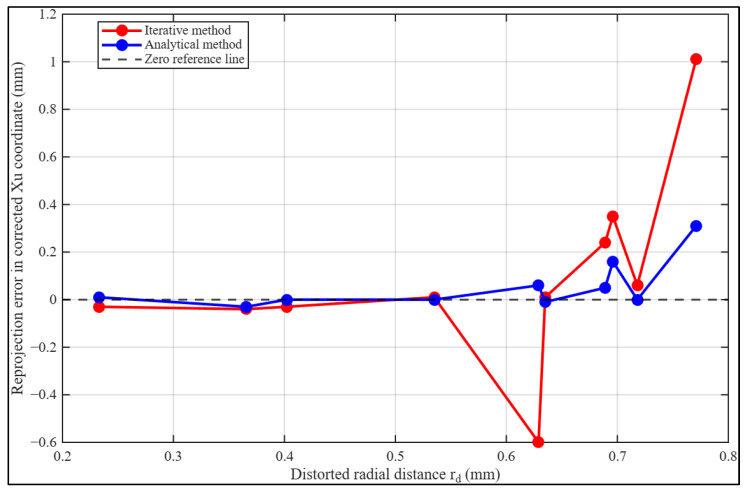
Reprojection error in the corrected $x_{u}$ coordinate as a function of distorted radius $r_{d}$ for the iterative and proposed analytical inversion methods.

Figure 4 presents a graphical comparison of the reprojection error from both methods in the $x_{u}$ direction against the distorted radius $r_{d}$. The analytical inversion maintains consistently low reprojection errors across the examined radial domain, remaining near zero even at higher distortion levels. The minimal divergence observed beyond approximately $r_{d}>0.63\mathrm{mm}$ underscores the greater numerical stability and robustness of the proposed analytical inversion strategy under stronger nonlinear distortion conditions. In contrast, the iterative approach begins to diverge beyond approximately $r_{d}>0.53\;\mathrm{mm}$, with errors increasing in magnitude and exhibiting greater variability. This divergence reflects the method’s sensitivity to stronger nonlinear distortion effects at larger radii, where higher-order polynomial terms dominate and approximation or truncation errors are amplified.

**Figure 5:**
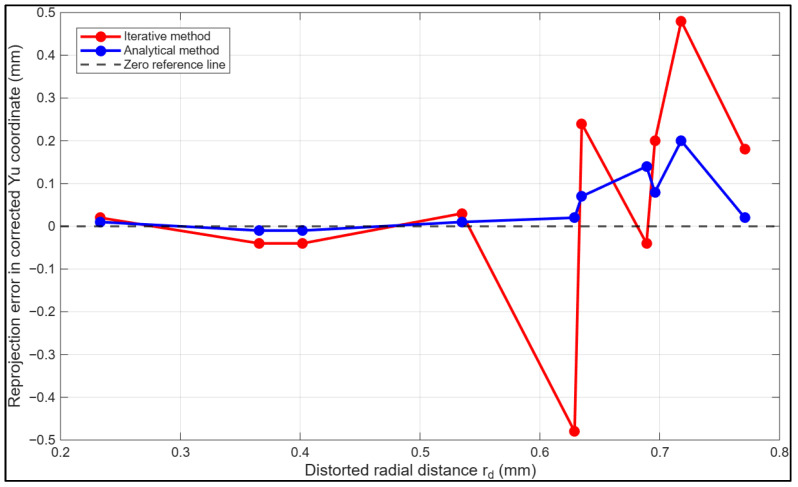
Reprojection error in the corrected $y_{u}$ coordinate as a function of distorted radius $r_{d}$ for the iterative and proposed analytical inversion methods.

Figure 5 represents the reprojection error in the corrected $y_{u}$ coordinate versus distorted radius $r_{d}$ for both calibration approaches. Similar to Figure 4, the analytical method demonstrates consistently reduced error magnitude and improved stability across all radii, with the error remaining below 0.2 mm. In contrast, the iterative approach shows pronounced error amplification at higher distortion levels, with the largest vertical error deviation observed at approximately 0.63 mm radius as well as more pronounced numerical instabilities beyond 0.63 mm radial distortion radius. This confirms the superior convergence behaviour of the proposed analytical formulation.

To further evaluate the effectiveness of the inverse radial distortion calibration algorithms, we computed the relative residual error for each corrected image point, following the formulation proposed by [36]:

(40) $$\mathrm{Relative}\;\mathrm{residual}\;\mathrm{error}=\frac{\gamma_{reference}-\gamma_{computed}}{\gamma_{reference}}$$

where $\gamma_{\mathrm{reference}}$ represents the measured distortion-free coordinate and $\gamma_{\mathrm{computed}}$ is the corresponding coordinate obtained through the inverse radial distortion models.

Table 10 summarizes the residual errors obtained after applying the iterative and proposed analytical inversion strategies to the radial distortion model over a distorted radius range of 0.233–0.771 mm. The results indicate that the analytical formulation consistently yields smaller residuals than the iterative approach across most tested radii. The iterative method shows more noticeable fluctuations in both the sign and magnitude of the residuals, especially at larger distorted radii (e.g., 0.718 mm and 0.771 mm), where the residuals increase to as much as ±0.119 mm in $y_{u}$ and ±0.064 mm in $x_{u}$.

In contrast, the analytical approach maintains lower dispersion, with maximum observed residuals of 0.044 mm in $x_{u}$ and 0.031 mm in $\;y_{u}$, and several cases showing near-zero values. Peak residuals observed in the iterative solution (e.g., ±0.119 mm in Y_u_) are significantly reduced in the analytical formulation (typically ≤0.031 mm). This improved stability is especially evident at larger distorted radii, where distortion effects are more pronounced.

**Figure 6:**
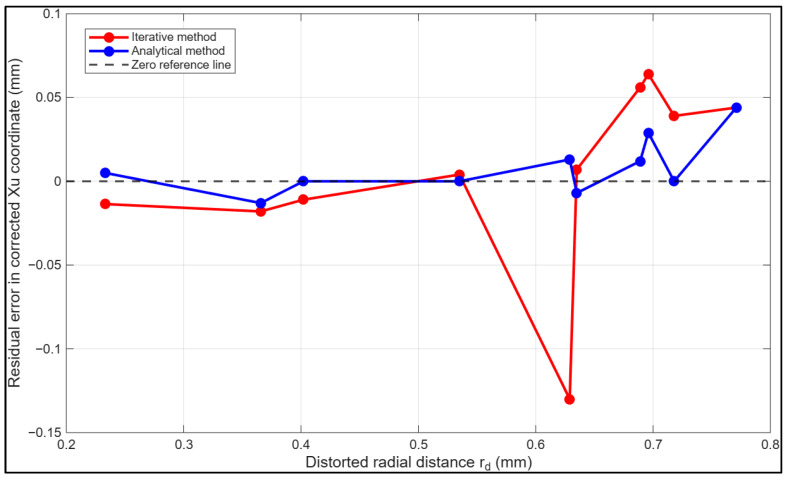
Comparison of residual errors for the corrected $x_{u}$ image coordinates. Residuals obtained from the iterative correction (red) are plotted against those from fully analytical calibration (blue). A zero-reference line (grey) is included to highlight deviations.

Figure 6 illustrates the residual errors associated with radial distortion correction using both the iterative and fully analytical strategies. The iterative method produces larger residuals, with peak deviations of −0.13 mm at approximately 0.53 mm radial distance and 0.064 mm at approximately 0.70 mm. In contrast, the fully analytical approach keeps residuals near zero, ranging from −0.013 mm at 0.233 mm to 0.044 mm at 0.70 mm radial distance, reflecting more accurate and consistent distortion correction across the image field.

The most pronounced differences between the two methods occur near the image periphery. For example, at approximately 0.63 mm radial distance, the iterative residual of −0.13 mm contrasts sharply with the analytical residual of 0.013 mm, corresponding to a difference of 0.143 mm. Similarly, at 0.70 mm, the discrepancy increases to 0.035 mm. Across almost the entire radial domain, the fully analytical method consistently reduces both the magnitude and variability of residuals, highlighting its superior capability to compensate for systematic distortions and preserve geometric fidelity, particularly in regions where lens distortions are most pronounced.

**Figure 7:**
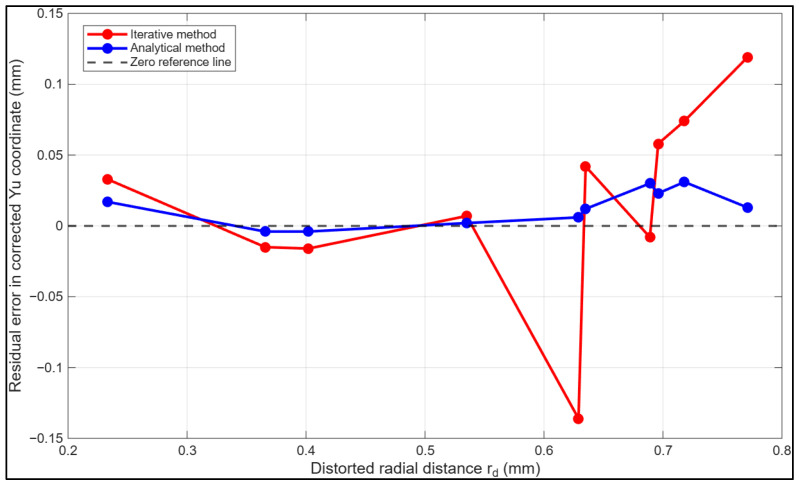
Comparison of residual errors for the corrected $y_{u}$ image coordinates. Residuals obtained from the iterative correction (red) are plotted against those from fully analytical calibration (blue). A zero-reference line (grey) is included to highlight deviations.

Figure 7 presents the residual errors for the corrected $y_{u}$ image coordinates using the iterative and fully analytical strategies. The iterative method exhibits larger fluctuations, with residuals varying from −0.136 mm at around 0.63 mm radial distance to 0.119 mm at 0.771 mm. In contrast, the fully analytical approach produces residuals that remain tightly constrained, ranging from −0.004 mm at 0.366 mm to 0.031 mm at 0.689 mm, reflecting a more accurate and consistent correction of distortions across the image field. The greatest differences between the two methods appear near the edges of the image. At approximately 0.63 mm, the iterative residual of −0.136 mm contrasts with the analytical residual of 0.006 mm, while at 0.771 mm the difference reaches 0.106 mm (0.119 mm versus 0.013 mm). Across most of the radial domain, the fully analytical method consistently yields smaller residuals with reduced variability, offering improved compensation of lens distortions and enhanced geometric fidelity, particularly in regions where distortion effects are strongest.

Table 10 summarizes the effectiveness of radial barrel distortion correction for each image point by comparing the corrected coordinates with their corresponding reference values. While these point-wise results provide detailed information, they do not capture the overall performance of each calibration algorithm across the full dataset. To evaluate this, we calculated the root mean square error (RMSE) relative to independently measured distortion-free reference coordinates [37]. Unlike the conventional RMS, which considers deviations from the mean of the computed coordinates, this approach measures errors with respect to reference values, providing a more accurate assessment for data distributed irregularly across the image. The root mean square errors associated with the x- and y-coordinate estimates are computed as follows:

(41) $$RMSE_{\gamma_{u}}=\sqrt{\frac{1}{T}\times \sum_{1}^{T}{\left(x_{\mathrm{reference}}-x_{\mathrm{computed}}\right)}^{2}}$$

(42) $$RMSE_{y_{u}}=\sqrt{\frac{1}{T}\times \sum_{1}^{T}{\left(y_{\mathrm{reference}}-y_{\mathrm{computed}}\right)}^{2}}$$

The overall RMSE is computed from (41) and (42) as follows:

(43) $$RMSE=\sqrt{{\left(RMSE_{x_{u}}\right)}^{2}+{\left(RMSE_{y_{u}}\right)}^{2}}$$

where T is the total number of image points selected in the image of the calibration pattern. To further extend the performance evaluation, the proposed analytical inverse model was benchmarked against both the iterative inverse approach and the classical Lagrange standard series reversion method. For the iterative inverse model, the RMSE along the $x_{u}$ and $y_{u}$ axes was 0.396 mm (≈1.50 pixels) and 0.244 mm (≈0.9 pixels), respectively, yielding an overall RMSE of 0.47 mm (≈1.78 pixels) as presented in Table 11. These values indicate substantial residual distortion, highlighting the limitations of the iterative approach in correcting severe barrel distortions. The Lagrange standard method exhibited even larger residual errors, with RMSE values of 0.378 mm and 0.585 mm along the $x_{u}$ and $y_{u}$ axes, respectively, producing an overall RMSE of 0.696 mm (≈2.63 pixels). The comparatively high error in the $y_{u}$ component suggests reduced numerical stability and diminished corrective capability when the distortion polynomial reaches higher orders. This behavior reflects the inherent truncation sensitivity of classical series reversion techniques under strong nonlinear distortion conditions. In contrast, the proposed analytical inverse model achieved substantially lower residual errors, with RMSE values of 0.114 mm and 0.085 mm along the $x_{u}$ and $y_{u}$ axes, respectively, and an overall RMSE of 0.142 mm (≈0.54 pixels). The reduction in total RMSE is significant when compared to both alternative methods, demonstrating superior numerical robustness and correction accuracy.

**Figure 8:**
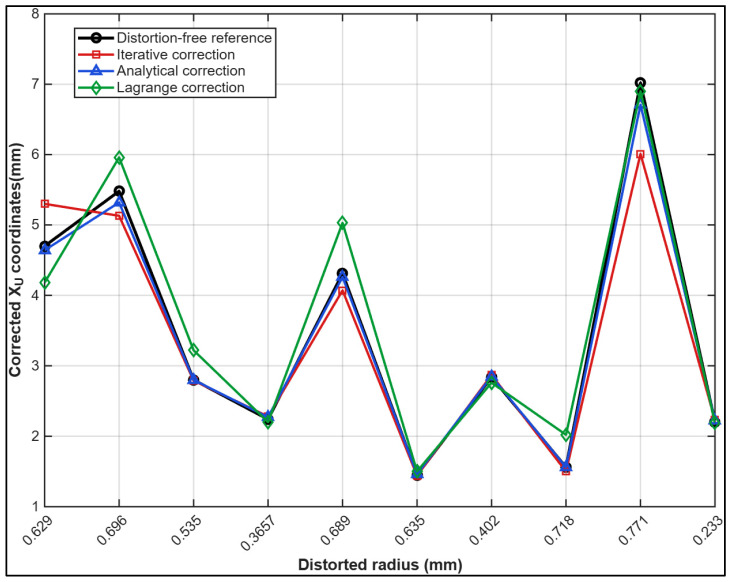
Comparison of corrected $x_{u}$ coordinates obtained with the iterative, analytical, and Lagrange reversion methods against measured distortion-free reference values, as function of radial distance.

Figure 8 demonstrates that both the iterative and analytical correction approaches closely reproduce the distortion-free $x_{u}$ coordinates across the full range of radial distances. The analytical solution consistently shows excellent agreement with the reference values, exhibiting only minor deviations even at larger radial distances, where distortion effects are typically more pronounced. The iterative method also performs well, although discrepancies increase slightly at higher radii. The Lagrange reversion method exhibits more variable behavior: it approximates the reference values reasonably well at small and intermediate radii but systematically underestimates $x_{u}$ coordinates at higher radial distances, with deviations increasing toward the periphery.

**Figure 9:**
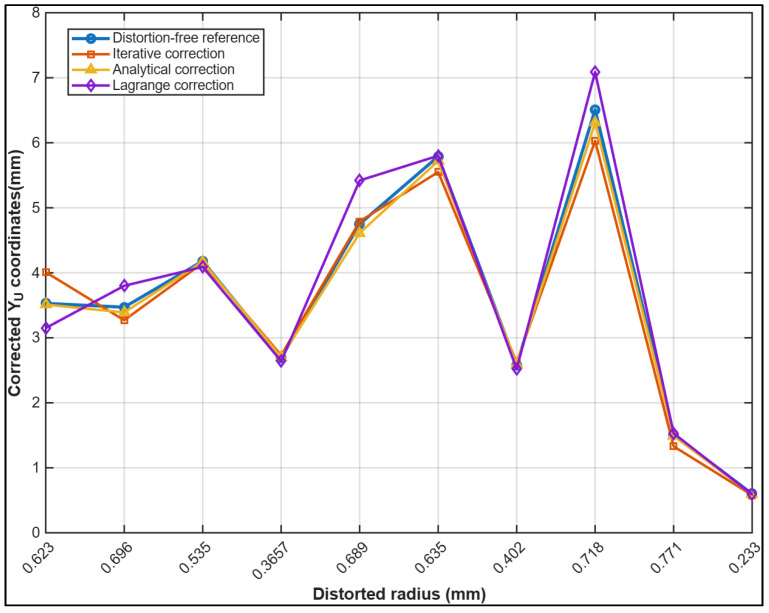
Comparison of corrected $y_{u}$ coordinates from the iterative, analytical, and Lagrange methods compared to measured distortion-free reference values as a function of radial distance.

The results for the $y_{u}$ coordinate correction in Figure 9 exhibit trends similar to those observed for the $x_{u}$ component. Both the analytical and iterative approaches demonstrate good agreement with the distortion-free reference data, maintaining stable accuracy across the full range of radial distances. The analytical correction appears slightly more consistent, particularly at larger radial distance values where distortion effects are strongest. The Lagrange reversion method again shows increased deviations at higher radial distances, underestimating coordinates near the periphery. These results confirm that the analytical and iterative approaches offer more reliable recovery of undistorted image coordinates, while the Lagrange method demonstrates reduced robustness under stronger distortion conditions.

## 4. Discussion

An analysis of the experimental results reported in Table 6, Table 7, Table 8, Table 9, Table 10 and Table 11 and Figure 2, Figure 3, Figure 4, Figure 5, Figure 6, Figure 7, Figure 8 and Figure 9 demonstrates that analytically derived inverse radial distortions coefficients provide a robust and accurate solution for correcting severe barrel distortion. The proposed analytical approach consistently produced substantially lower reprojection errors than both the iterative strategy and the Lagrange standard inversion method, particularly when high-order polynomial models are required to represent complex distortion profiles.

Severe barrel distortion profile was effectively represented using a depressed eighth-degree polynomial model, and its inverse was accurately recovered using the proposed analytical framework. As shown in the seventh row of Table 9, the analytical approach achieved an average reprojection error of 0.09 mm (≈0.036 pixels), which lies well within acceptable limits for close-range photogrammetry and three-dimensional reconstruction. In contrast, the iterative method resulted in reprojection errors exceeding 0.6 pixels, indicating limited capability in recovering a stable inverse mapping for this class of distortion.

The Lagrange series reversion method yielded comparatively larger residuals, with reprojection errors of −0.136 mm (≈0.51 pixels) in the $x_{u}$ direction and 0.43 mm (≈1.63 pixels) in the $y_{u}$ direction. The negative sign in the $x_{u}$ component indicates a slight overestimation of the distortion-free reference coordinate, reflecting a systematic bias; however, performance evaluation is governed by error magnitude rather than sign. The global behaviour is confirmed by the RMSE results in Table 11, where the Lagrange method records an overall error of approximately 2.63 pixels, exceeding both the iterative and analytical approaches. These findings demonstrate that Lagrange reversion lacks the numerical stability and precision achieved by the proposed analytical inverse framework.

The disparity in performance among the three approaches underscores the limitations of both iterative and classical series reversion techniques when applied to high-degree radial distortion models. The iterative method exhibits limited robustness under severe barrel distortion, producing residual errors that exceed acceptable photogrammetric thresholds. The Lagrange series reversion, while providing a formally derived polynomial inverse, demonstrates even greater sensitivity to truncation effects and coefficient coupling. In high-order models, these effects become increasingly pronounced at larger radial distances, leading to amplified residual deviations and peripheral over- or under-corrections.

By contrast, the proposed analytical inversion framework demonstrates strong numerical stability across all evaluated distortion scenarios. The direct derivation of the inverse polynomial coefficients eliminates iterative dependency and substantially reduces parameter coupling, thereby limiting the propagation and amplification of high-order estimation errors. This structural formulation enables consistent suppression of residual barrel distortion without inducing secondary artifacts such as unintended pincushion effects, resulting in a more faithful recovery of distortion-free image coordinates.

Overall, the results confirm that fully analytical inverse modeling is essential for reliably correcting severe barrel radial distortions represented by high-degree polynomial functions. The proposed framework achieves substantially lower reprojection errors and superior global stability compared to both iterative approximation and Lagrange series reversion. While classical series inversion offers a mathematically valid expansion, its practical performance degrades in the presence of strong distortion nonlinearity. The analytical formulation, by contrast, provides improved accuracy, robustness, and predictability, making it particularly well suited for demanding photogrammetric and computer vision applications requiring high geometric fidelity.

Table 12 highlights key advantages of the proposed analytical method over the [33] approach and Lagrange standard inversion method for computing inverse coefficients of polynomial radial distortion models.

Unlike the iterative and recursive formulation presented in [32], which may experience convergence instability and error accumulation when applied to high-degree polynomial models, both the Lagrange series reversion and the proposed analytical framework operate in a non-iterative, non-recursive manner. Nevertheless, the Lagrange expansion remains sensitive to truncation order and coefficient coupling effects. These sensitivities become increasingly pronounced at larger radial distances, where higher-order terms dominate, leading to amplified residual deviations and moderate reprojection errors.

In contrast, the proposed analytical inversion strategy is based on the direct derivation of inverse polynomial coefficients, thereby avoiding recursive dependency and iterative convergence issues. This coefficient-level formulation enhances numerical stability, limits parameter interaction effects, and reduces the risk of high-order error amplification. As a result, the method reliably handles high-degree depressed polynomial distortion models, effectively suppresses residual barrel distortion without introducing secondary artifacts, and demonstrates reduced sensitivity to reprojection error propagation. By comparison, the approach in [32] exhibits practical limitations when extended to high-degree depressed polynomials, where recursive computation can increase susceptibility to instability and error amplification.

Table 13 compares the calibration performance of the proposed method with selected state-of-the-art techniques using root mean square error as the evaluation metric. To en-sure a fair comparison, all approaches employed checkerboard-based calibration patterns to extract image point coordinates.

The method reported by [38] demonstrated the highest root mean square (RMS) error among the evaluated techniques, indicating comparatively limited suitability for applications requiring high-precision geometric correction. Although the approach effectively integrates radial distortion compensation with infrared radiometric nonlinearity correction, its geometric accuracy remains less competitive in purely distortion-focused assessments.

Improved performance was observed in the calibration strategy presented in [39], which achieved an RMS error of approximately 0.9 pixels. Further refinement is evident in the light-field-based method proposed by [40]. Light field endoscope calibration based on virtual objective lens and virtual feature points, where the reported RMS error of about 0.8 pixels satisfies the accuracy requirements of many photogrammetric and computer vision applications. Among the reviewed studies, the concentric-circle compensation approach introduced by [41] based on concentric circle compensation for industrial environments delivered particularly strong performance, yielding the second-lowest calibration error of approximately 0.7 pixels and demonstrating robustness under industrial imaging conditions.

In contrast, the analytical framework proposed in this study, based on explicit estimation of inverse radial distortion coefficients achieved a substantially lower average RMS error of approximately 0.54 pixels. This improvement underscores the advantage of directly modeling the inverse distortion function, rather than relying on forward polynomial compensation or iterative correction schemes. By analytically deriving the inverse distortion relationship, the proposed method reduces residual reprojection error and enhances numerical stability. The results therefore indicate that inverse-coefficient-based correction offers a more precise and robust solution for radial distortion calibration, outperforming conventional compensation approaches commonly implemented in existing distortion correction software.

## 5. Conclusions

A fundamental limitation of traditional radial distortion models lies in the difficulty of computing reliable inverse mappings, which has historically constrained calibration accuracy. Recursive and iterative inversion techniques, although widely adopted, lack direct coefficient-level derivations and depend on convergence-based refinement. Such approaches are computationally demanding and susceptible to instability, particularly for high-degree polynomial models where error accumulation and parameter coupling can significantly degrade performance. Furthermore, their inverse formulations are often model-specific and inflexible, limiting adaptability across varying distortion structures.

To overcome these challenges, this study introduced an analytical coefficient-based framework for radial distortion inversion that enables direct derivation of inverse polynomial coefficients without iterative optimization. By reformulating the inversion problem within a constrained algebraic structure, the proposed method establishes a general mechanism for constructing inverse radial distortion models for polynomials of arbitrary degree. This formulation reduces recursive dependency, mitigates coefficient interaction effects, and enhances numerical stability across a wide spectrum of distortion scenarios.

Experimental evaluation demonstrated that the proposed inverse-coefficient-based calibration strategy consistently achieves substantially lower reprojection and calibration errors than existing recursive, iterative, and series-based approaches. The method proved particularly effective for high-order distortion models, including depressed eighth-degree polynomials, which are known to challenge conventional inversion techniques. The elimination of iterative refinement not only improves numerical robustness but also reduces computational overhead, making the framework suitable for high-precision photogrammetric and computer vision applications.

The Lagrange series reversion method was evaluated as a classical non-iterative benchmark. Although it provides a formally derived polynomial inverse approximation, its practical performance deteriorates for high-degree models due to truncation sensitivity and coefficient coupling effects. This behaviour results in amplified residual errors, particularly toward peripheral image regions, and higher overall reprojection error compared to both the iterative and proposed analytical strategies. While Lagrange reversion remains mathematically valid, it does not offer the numerical stability required for severe distortion conditions.

Beyond numerical performance, the proposed framework contributes a unified algebraic perspective on inverse radial distortion modeling. Unlike traditional inversion techniques that rely on approximate expansions or iterative refinement, the method systematically derives inverse profiles that adapt to different polynomial structures and distortion characteristics. This generality enhances robustness and provides a consistent foundation for modeling diverse radial distortion behaviours within a single coefficient-based formulation.

Improved distortion correction accuracy has important downstream implications. Reduced reprojection error directly limits geometric error propagation in tasks such as camera pose estimation, three-dimensional reconstruction, and precision measurement extraction. These improvements are particularly critical in applications where small geometric deviations can cascade into substantial downstream inaccuracies, including photogrammetry, geospatial analysis, and industrial metrology. Consequently, the proposed method strengthens the reliability of camera-based measurement systems operating in precision-sensitive domains.

Future research may extend the analytical coefficient derivation framework to alternative lens models, including fisheye and panoramic systems exhibiting more extreme distortion characteristics. The explicit computation of parameter-specific inverse coefficients also presents opportunities for hybrid integration with data-driven techniques. Machine learning models could leverage prior calibration datasets to accelerate parameter estimation under varying imaging conditions, while adaptive systems such as robotic vision platforms may benefit from real-time analytical inversion capabilities.

Further investigation into polynomial reduction strategies, including transformations such as Tschirnhaus-based reformulations, may enhance computational efficiency while preserving inversion accuracy. Refinement of such techniques could yield additional gains in applications where even marginal improvements in geometric precision are operationally significant.

Overall, the analytical inversion framework established in this study provides a robust, extensible, and numerically stable foundation for high-degree radial distortion correction. By outperforming recursive, iterative, and classical series reversion methods in accuracy and stability, it advances the state of inverse polynomial calibration and supports precision-critical imaging applications.

## Figures and Tables

**Table 1:** Characteristics of the Xiaomi Redmi mobile phone under the ultra-wide-angle mode.

Optical Image Acquisition Device	Characteristics
Image sensor model	Sony IMX 355 CMOS sensor
Native sensor resolution	108 MP
Wide-angle effective resolution	8 MP
Highest supported pixel dimensions	2400 × 1080 px
Active sensor area	6.058 × 4.415 mm^2^
Pixel pitch	0.7 μm
Optical focal length	0.026 m
Image storage format	jpeg

**Table 2:** Comparative analysis of inverse radial distortion coefficients obtained using the iterative and fully analytical methodologies.

Inverse Coefficients	Formulations of Iterative Inversion Algorithms	Formulations of Analytical Inversion Algorithms
$\omega_{1}$	$-\lambda_{1}$	$-\lambda_{1}$
$\omega_{2}$	$3\lambda_{1}^{2}-\lambda_{2}$	$-\lambda_{1}^{3}-\lambda_{2}$
$\omega_{3}$	$-12\lambda_{1}^{3}+8\lambda_{1}\lambda_{2}-\lambda_{3}$	$-2\lambda_{1}^{5}-8\lambda_{1}^{2}\lambda_{2}-\lambda_{3}$
$\omega_{4}$	$55\lambda_{1}^{4}+10\lambda_{1}\lambda_{3}-55\lambda_{1}^{2}\lambda_{2}+5\lambda_{2}^{2}-\lambda_{4}$	$-5\lambda_{1}^{7}-17\lambda_{1}^{2}\lambda_{3}-31\lambda_{1}^{4}\lambda_{2}-13\lambda_{1}\lambda_{2}^{2}-\lambda_{4}$

**Table 3:** Radial distortion coefficients computed using MATLAB’s camera calibration procedure.

Radial Distortion Terms	Numerical Coefficient Values
$\lambda_{1}$	$-1.936\times 10^{-1}$
$\lambda_{2}$	$3.8\times 10^{-3}$
$\lambda_{3}$	$3.38\times 10^{-2}$
$\lambda_{4}$	0.00

**Table 4:** Estimated Inverse Polynomial Coefficients for Radial Lens Distortion Correction Using an Eighth-Order Distortion Model.

Forward Radial Distortion Coefficients	Inverse Radial Distortion Coefficients	Iterative Determination of Radial Distortion Coefficients	Fully Analytical Determination of Radial Distortion Coefficients
$-1.936\times 10^{-1}$	$\omega_{1}$	$1.936\times 10^{-1}$	$1.936\times 10^{-1}$
$0.038\times 10^{-1}$	$\omega_{2}$	0.1086	0.00342
$0.338\times 10^{-1}$	$\omega_{3}$	0.047395	−0.034395
0.00	$\omega_{4}$	0.004068	−0.00216928

**Table 5:** Corrected $x_{u}$ and $y_{u}$ coordinates compared with their corresponding measured distortion-free reference $x_{u}$ and $y_{u}$ coordinates.

Reference Distortion Free Coordinates		Undistorted Data Points Computed Using the Iteratively Derived Inverse Model		Undistorted Data Points Computed Using Analytically Derived Inverse Model	
$x_{u}$	$y_{u}$	$x_{u}$	$y_{u}$	$x_{u}$	$y_{u}$
4.70	3.53	5.30	4.01	4.64	3.51
5.48	3.47	5.13	3.27	5.32	3.39
2.80	4.18	2.79	4.15	2.80	4.17
2.24	2.69	2.28	2.73	2.27	2.70
4.31	4.75	4.07	4.79	4.26	4.61
1.45	5.79	1.44	5.55	1.46	5.72
2.84	2.58	2.87	2.62	2.85	2.59
1.56	6.51	1.50	6.03	1.56	6.31
7.02	1.51	6.01	1.33	6.71	1.49
2.20	0.60	2.23	0.58	2.22	0.59

**Table 6:** Distribution of a subset of corrected distortion-free $x_{u}$ coordinates relative to the interval bounded by the reference undistorted measurements and their corresponding distorted values.

$\mathrm{Reference}\;\mathrm{Measured}\;\mathrm{Distortion}\text{-}\mathrm{Free}\;x_{u}$ Coordinates	$\mathrm{Corrected}\;\mathrm{Distortion}\text{-}\mathrm{Free}\;x_{u}$ Coordinates Using the Iterative Method	$\mathrm{Corrected}\;\mathrm{Distortion}\text{-}\mathrm{Free}\;x_{u}$ Coordinates Using the Analytical Method	$\mathrm{Measured}\;\mathrm{Distorted}\;x_{d}$ Reference Coordinates
4.70	5.30	4.64	5.02
5.48	5.13	5.32	5.87
2.80	2.79	2.80	2.98
2.24	2.28	2.27	2.34
4.31	4.07	4.26	4.63
1.45	1.44	1.46	1.60
2.84	2.87	2.84	2.97
1.56	1.50	1.56	1.73
7.02	6.01	6.71	7.53
2.20	2.23	2.19	2.25

**Table 7:** Distribution of a subset of corrected distortion-free $y_{u}$ coordinates relative to the interval bounded by the reference undistorted measurements and their corresponding distorted values.

$\mathrm{Reference}\;\mathrm{Measured}\;\mathrm{Distortion}\;\mathrm{Free}\;y_{u}$ Coordinates	$\mathrm{Computed}\;\mathrm{Distortion}\;\mathrm{Free}\;y_{u}$ Coordinates Using Iteratively Derived Method	$\mathrm{Computed}\;\mathrm{Distortion}\;\mathrm{Free}\;y_{u}$ Coordinates Using Analytically Derived Method	$\mathrm{Reference}\;\mathrm{Measured}\;\mathrm{Distorted}\;y_{d}$ Coordinates
3.53	4.01	3.51	3.79
3.47	3.27	3.39	3.74
4.18	4.15	4.17	4.44
2.69	2.73	2.70	2.81
4.75	4.79	4.61	5.10
5.79	5.55	5.72	6.17
2.58	2.62	2.59	2.71
6.51	6.03	6.31	6.97
1.51	1.33	1.49	1.67
0.60	0.58	0.59	0.61

**Table 8:** Computed reprojection errors for a subset of points after applying radial distortion corrections per calibration method.

Reprojection Errors for the Iterative Algorithm in mm		Reprojection Errors for the Analytical Algorithm in mm	
$x_{u}$	$y_{u}$	$x_{u}$	$y_{u}$
−0.6	−0.48	0.06	0.02
0.35	0.2	0.16	0.08
0.01	0.03	0	0.01
−0.04	−0.04	−0.03	−0.01
0.24	−0.04	0.05	0.14
0.01	0.24	−0.01	0.07
−0.03	−0.04	0	−0.01
0.06	0.48	0	0.2
1.01	0.18	0.31	0.02
−0.03	0.02	0.01	0.01

**Table 9:** Reprojection error (mm) per corrected coordinates per calibration strategy.

Distorted Radius	Iterative	Analytical	Iterative	Analytical
$r_{d}$	$x_{u}$	$x_{u}$	$y_{u}$	$y_{u}$
0.629	−0.60	0.06	−0.48	0.02
0.696	0.35	0.16	0.20	0.08
0.535	0.01	0.00	0.03	0.01
0.3657	−0.04	−0.03	−0.04	−0.01
0.689	0.24	0.05	−0.04	0.14
0.635	0.01	−0.01	0.24	0.07
0.402	−0.03	0.00	−0.04	−0.01
0.718	0.06	0.00	0.48	0.20
0.771	1.01	0.31	0.18	0.02
0.233	−0.03	0.01	0.02	0.01

**Table 10:** Residual reconstruction errors (mm) in the corrected coordinates for each calibration strategy across a representative range of distorted radii (0.233–0.771 mm).

Distorted Radius	Iterative	Analytical	Iterative	Analytical
$r_{d}$	$x_{u}$	$x_{u}$	$y_{u}$	$y_{u}$
0.629	−0.130	0.013	−0.136	0.006
0.696	0.064	0.029	0.058	0.023
0.535	0.004	0.000	0.007	0.002
0.3657	−0.018	−0.013	−0.015	−0.004
0.689	0.056	0.012	−0.008	0.030
0.635	0.007	−0.007	0.042	0.012
0.402	−0.011	0.000	−0.016	−0.004
0.718	0.039	0.000	0.074	0.031
0.771	0.044	0.044	0.119	0.013
0.233	−0.014	0.005	0.033	0.017

**Table 11:** Summary of RMSE values obtained using different calibration approaches.

Radial Distortion Correction Algorithms	${\mathrm{RMSE}}_{x_{u}}$	${\mathrm{RMSE}}_{y_{u}}$	RMSE (mm)	RMSE (pixel)
Iterative	0.396	0.244	0.465	1.8
Lagrange Standard method	0.378	0.585	0.696	2.63
Fully analytical	0.114	0.085	0.142	0.537

**Table 12:** Summary of advantages of the newly developed radial distortion polynomial inversion strategy.

Features	[32]	Lagrange Series Reversion	Proposed Analytical Method
Iterative?	Yes	No	No
Recursive?	Yes	No	No
Handles high-degree polynomials	Limited (unstable)	Moderate (truncation sensitive)	Yes (stable)
Symbolic/Analytical solution	Partially	Partially derived	Fully
Reprojection error sensitivity	High	Moderate	Low

**Table 13:** Comparison of radial distortion correction root mean square error in pixel per calibration method.

Ref. [38]	Ref. [39]	Ref. [40]	Ref. [41]	New Fully Analytical Method
1.6	0.9	0.8	0.7	0.54

## Data Availability

The data presented in this study are available on request from the corresponding author because it is part of a research laboratory data repository.

## Appendix A. Structural Analysis of Table A1: High-Order Inverse Radial Distortion Coefficients

Table A1 lists the first seven inverse radial distortion coefficients derived using three inversion strategies: the recursive/iterative formulation [32], the Lagrange standard series reversion, and the proposed analytical coefficient derivation framework. The explicit expressions reveal systematic structural differences in coefficient scaling, power dependence, and dynamic range growth.

**Table A1:** General Iterative and Analytical Inverse Formulas for High-Order Radial Distortion Polynomials.

Original Coefficients	Iteratively Reversed Coefficients	Lagrange Standard Series Reversion	Proposed Analytically Reversed Coefficients
$\kappa_{1}$	$-\kappa_{1}$	$k_{1}^{-1}$	$-\kappa_{1}$
$\kappa_{2}$	$3\kappa_{1}^{2}-\kappa_{2}$	$-\kappa_{1}^{-3}\kappa_{2}$	$-2\kappa_{1}^{2}-\kappa_{2}$
$\kappa_{3}$	$-12\kappa_{1}^{3}+8\kappa_{1}\kappa_{2}-\kappa_{3}$	$\kappa_{1}^{-5}\left(2\kappa_{2}^{2}-\kappa_{1}\kappa_{3}\right)$	$-\kappa_{1}^{3}-5\kappa_{1}\kappa_{2}-\kappa_{3}$
$\kappa_{4}$	$-55\kappa_{1}^{2}\kappa_{2}+5\kappa_{2}^{2}+10\kappa_{1}\kappa_{3}+55\kappa_{1}^{4}-\kappa_{4}$	$\kappa_{1}^{-7}\left(5\kappa_{1}\kappa_{2}\kappa_{3}-\kappa_{1}^{2}\kappa_{4}-5\kappa_{2}^{3}\right)$	$-5\kappa_{1}^{2}\kappa_{2}-3\kappa_{2}^{2}-6\kappa_{1}\kappa_{3}-\kappa_{4}$
$\kappa_{5}$	$\begin{array}{l} 364\kappa_{1}^{3}\kappa_{2}-78\kappa_{1}\kappa_{2}^{2}-78\kappa_{1}^{2}\kappa_{3}+12\kappa_{2}\kappa_{3}\\ +12\kappa_{1}\kappa_{4}-273\kappa_{1}^{5} \end{array}$	$\kappa_{1}^{-9}\left(6\kappa_{1}^{2}\kappa_{2}\kappa_{4}+3\kappa_{1}^{2}\kappa_{3}^{2}+14\kappa_{2}^{4}-\kappa_{1}^{3}\kappa_{5}-21\kappa_{1}\kappa_{2}^{2}\kappa_{3}\right)$	$\begin{array}{l} -\kappa_{1}^{3}\kappa_{2}-7\kappa_{1}\kappa_{2}^{2}-8\kappa_{1}^{2}\kappa_{3}-7\kappa_{2}\kappa_{3}\\ -7\kappa_{1}\kappa_{4}-\kappa_{5} \end{array}$
$\kappa_{6}$	$\begin{array}{l} 840\kappa_{1}^{2}\kappa_{2}^{2}-35\kappa_{2}^{3}+560\kappa_{1}^{3}\kappa_{3}\\ -210\kappa_{1}\kappa_{2}\kappa_{3}+7\kappa_{3}^{2}-105\kappa_{1}^{2}\kappa_{4}\\ +14\kappa_{2}\kappa_{4}+1428\kappa_{1}^{6}-2380\kappa_{1}^{4}\kappa_{2} \end{array}$	$\kappa_{1}^{-11}\left(\begin{array}{c} 7\kappa_{1}^{3}\kappa_{2}\kappa_{5}+7\kappa_{1}^{3}\kappa_{3}\kappa_{4}+84\kappa_{1}\kappa_{2}^{3}\kappa_{3}\\ -\kappa_{1}^{4}\kappa_{6}-28\kappa_{1}^{2}\kappa_{2}\kappa_{3}^{2}-42\kappa_{2}^{5}-28\kappa_{1}^{2}\kappa_{2}^{2}\kappa_{4} \end{array}\right)$	$\begin{array}{l} -3\kappa_{1}^{2}\kappa_{2}^{2}-3\kappa_{2}^{3}-4\kappa_{1}^{3}\kappa_{3}-20\kappa_{1}\kappa_{2}\kappa_{3}-4\kappa_{3}^{2}\\ -12\kappa_{1}^{2}\kappa_{4}-8\kappa_{2}\kappa_{4}-8\kappa_{1}\kappa_{5}-\kappa_{6} \end{array}$
$\kappa_{7}$	$\begin{array}{l} -3876\kappa_{1}^{4}\kappa_{3}+816\kappa_{1}\kappa_{2}^{3}+2448\kappa_{1}^{2}\kappa_{2}\kappa_{3}\\ -136\kappa_{2}^{2}\kappa_{3}-136\kappa_{1}\kappa_{3}^{2}+816\kappa_{1}^{3}\kappa_{4}\\ -272\kappa_{1}\kappa_{2}\kappa_{4}+16\kappa_{3}\kappa_{4}+15504\kappa_{2}\kappa_{1}^{5}\\ -7752\kappa_{1}^{3}\kappa_{2}^{2}-7752\kappa_{1}^{7} \end{array}$	$\kappa_{1}^{-13}\left(\begin{array}{l} 8\kappa_{1}^{4}\kappa_{2}\kappa_{6}+8\kappa_{1}^{4}\kappa_{3}\kappa_{5}+4\kappa_{1}^{4}\kappa_{4}^{2}\\ +120\kappa_{1}^{2}\kappa_{2}^{3}\kappa_{4}+180\kappa_{1}^{2}\kappa_{2}^{2}\kappa_{3}^{2}\\ +132\kappa_{2}^{6}-\kappa_{1}^{4}\kappa_{7}-36\kappa_{1}^{3}\kappa_{2}^{2}\kappa_{5}\\ -72\kappa_{1}^{3}\kappa_{2}\kappa_{3}\kappa_{4}-12\kappa_{1}^{3}\kappa_{3}^{3}-330\kappa_{1}\kappa_{2}^{4}\kappa_{3} \end{array}\right)$	$\begin{array}{l} -\kappa_{1}^{4}\kappa_{3}-3\kappa_{1}\kappa_{2}^{3}-15\kappa_{1}^{2}\kappa_{2}\kappa_{3}-12\kappa_{2}^{2}\kappa_{3}\\ -13\kappa_{1}\kappa_{3}^{2}-10\kappa_{1}^{3}\kappa_{4}-28\kappa_{1}\kappa_{2}\kappa_{4}-9\kappa_{3}\kappa_{4}\\ -17\kappa_{1}^{2}\kappa_{5}-9\kappa_{1}\kappa_{6}-9\kappa_{2}\kappa_{5}-\kappa_{7} \end{array}$

### Appendix A.1. Power Dependence on $\kappa_{1}$

A fundamental distinction between the three formulations is the dependence of higher-order inverse coefficients on the first radial distortion parameter $\kappa_{1}$.

### Appendix A.2. Iterative Formulation

From Table A1, the dominant terms of orders 4–7 include:

$$55\kappa_{1}^{4},\;273\kappa_{1}^{5},1428\kappa_{1}^{6},7752\kappa_{1}^{7}$$

Thus, the nth-order coefficient contains a pure high-degree term:

$$a_{n}^{\left(iter\right)}∼C_{n}\kappa_{1}^{n}$$

where $C_{n}$ increases combinatorially. This produces polynomial growth whose magnitude escalates rapidly with order.

### Appendix A.3. Lagrange Series Reversion

The Lagrange column in Table A1 shows explicit reciprocal powers:

$$\kappa_{1}^{-3},\;\kappa_{1}^{-5},\kappa_{1}^{-7},\kappa_{1}^{-9},\;\kappa_{1}^{-11}$$

Hence,

$$a_{n}^{\left(Lag\right)}\propto \;\kappa_{1}^{-\left(2n-1\right)}$$

For calibrated cameras where $\left|\kappa_{1}\right|<1$, this produces exponential amplification of higher-order terms. For example, if $\kappa_{1}=-0.1$, then $\kappa^{-13}=10^{13}$, leading to severe dynamic range inflation.

### Appendix A.4. Proposed Analytical Framework

Inspection of Table A1 shows:

- No reciprocal powers of $\kappa_{1}$
- No pure high-degree terms $\kappa_{1}^{n}$ for large n.
- Highest observed power of $\kappa_{1}\leq 4\;\left(\mathrm{up}\;\mathrm{to}\;7\mathrm{th}\;\mathrm{order}\right)$
- Highest distortion coefficient $\kappa_{n}$ appears linearly

Thus,

$$a_{n}^{\left(prop\right)}-O\left(\kappa_{1}^{m}\right),\;m\leq 4$$

within the examined range. The growth rate is therefore bounded and does not scale directly with inversion order.

### Appendix A.5. Magnitude Escalation and Dominant-Term Ratios

The iterative coefficients show rapid multiplicative growth in dominant weights:

55→273→1428→7752

This super-linear escalation increases the dynamic range of successive coefficients and heightens sensitivity to rounding and cancellation effects.

In contrast:

- Lagrange escalation is driven by reciprocal powers $\kappa_{1}^{-2}$ per order increment.
- The proposed framework maintains comparatively stable leading-term magnitudes (≤30 in Table A1), preventing runaway amplification.

### Appendix A.6. High-Power Dominance and Cancellation Risk

The iterative coefficients contain competing large-magnitude terms, e.g.,

$$+15504\kappa_{1}^{5}\kappa_{2}-7752\kappa_{1}^{7}$$

Such opposing dominant contributions increase floating-point cancellation sensitivity, particularly at high radial distances where higher-order terms dominate.

The proposed analytical coefficients:

- Avoid pure high-degree dominance,
- Distribute influence across mixed lower-order products,
- Introduce each new distortion parameter linearly,
- Suppress combinatorial explosion.

### Appendix A.7. Conditioning Implications for Radial Distortion Inversion

From the structural patterns in Table A1:

- The Lagrange formulation exhibits reciprocal asymptotic divergence.
- The iterative formulation exhibits combinatorial polynomial escalation.
- The proposed framework demonstrates bounded polynomial growth.

These structural properties directly affect numerical conditioning when evaluating high-degree inverse distortion at large radii. The bounded growth behaviour of the proposed analytical formulation reduces amplification of parameter noise and improves robustness in practical calibration scenarios. The explicit coefficient expressions in Table A1 demonstrate that inversion strategies differ structurally rather than procedurally. The proposed analytical inversion framework uniquely:

- Eliminates reciprocal power dependence,
- Suppresses high-degree pure $\kappa_{1}^{n}$ dominance,
- Maintains bounded magnitude growth across successive orders,
- Reduces dynamic range escalation and cancellation sensitivity.

These properties explain the improved numerical stability and reduced reprojection error observed in the experimental section.

## References

[1] Zhang Y., Liu J., Zhao Y., Xu J., Wu W. (2025). Crack Detection on Concrete Composite Slab Using You Only Look Once Version 8 Based Lightweight System. J. Build. Eng..

[2] Rossi G. (2025). Analogic image formation. Mechanical and Thermal Measurements by Images and Waves: Principles and Applications.

[3] Zeng C., Wei R., Gu M., Zhang N., Dai Z. (2024). High-precision calibration method and error analysis of infrared binocular target ranging systems. Electronics.

[4] Ge P., Dan X., Wang H., Gao H., Wang Y., Li G. (2025). High-precision calibration method for the multiview measurement of the mirror–assisted digital image correlation. Measurement.

[5] Moru D.K., Borro D. (2021). Improving optical pipeline through better alignment and calibration process. Int. J. Adv. Manuf. Technol..

[6] Yang H.M., Hsu C.C., Lin P.C., Lin H.Y., Chen S.H., Cheng H.C., Fang W. (2024). Design of bi-axial piezoelectric MEMS micro mirror with gimbal actuator for dynamic decoupling. J. Micromech. Microeng..

[7] Huang M., Zheng B., Li R., Zou Y., Li X., Qian C., Lu H., Zhu R., Chen H. (2025). Real-time all-directional 3D recognition and multidistortion correction via prior diffraction neural networks. Adv. Photonics.

[8] Brown D.C. (1971). Close-range camera calibration. Photogramm. Eng..

[9] Fryer J.G., Brown D.C. (1986). Lens distortion for close-range photogrammetry. Photogramm. Eng. Remote Sens..

[10] Ma X., Zhu P., Li X., Zheng X., Zhou J., Wang X., Au K.W.S. (2024). A minimal set of parameters-based depth-dependent distortion model and its calibration method for stereo vision systems. IEEE Trans. Instrum. Meas..

[11] Chaudhry F.M., Ralli J., Leudet J., Sohrab F., Pakdaman F., Corbani P., Gabbouj M. (2025). Deep-BrownConrady: Prediction of camera calibration and distortion parameters using deep learning and synthetic data. arXiv.

[12] Cong Q.N., Choi S. (2025). Generalized camera calibration: Camera model selection and calibration with effective image sampling. IEEE Sens. J..

[13] Zhang Y., Song X., Guan X., Yu Y. (2023). Radial distortion correction method based on depth-separable network. Proceedings of the 2023 7th International Conference on Transportation Information and Safety (ICTIS), Xi’an, China, 4–6 August 2023.

[14] Leotta M.J., Russell D., Matrai A. On the maximum radius of polynomial lens distortion. Proceedings of the IEEE/CVF Winter Conference on Applications of Computer Vision.

[15] Liebold F., Mader D., Sardemann H., Eltner A., Maas H.G. (2023). A bi-radial model for lens distortion correction of low-cost UAV cameras. Remote Sens..

[16] Montibeller A., Pérez-González F. (2023). An adaptive method for camera attribution under complex radial distortion corrections. IEEE Trans. Inf. Forensics Secur..

[17] Janos I., Benesova W. (2024). Improving radial lens distortion correction with multi-task learning. Pattern Recognit. Lett..

[18] Yu J., Zhang Z., Sun H., Xia Z., Wen H. (2024). Reevaluating the underlying radial symmetry assumption of camera distortion. IEEE Trans. Instrum. Meas..

[19] Shi Q., Plancher E., Loisnard D., Karamched P., Liu J., Chen Z., Wang H., Roux S. (2022). Improved high-resolution EBSD analyses by correcting radial distortion of electron diffraction patterns. Mater. Charact..

[20] Kabbani W., Le Pessot T., Raja K., Ramachandra R., Busch C. (2024). Radial distortion in face images: Detection and impact. Proceedings of the 2024 IEEE International Joint Conference on Biometrics (IJCB), Zurich, Switzerland, 30 September–3 October 2024.

[21] Guo K., Ye H., Chen H., Gao X. (2022). A new method for absolute pose estimation with unknown focal length and radial distortion. Sensors.

[22] Wang Q., Cheng W.-C., Suresh N., Hua H. (2016). Development of the local magnification method for quantitative evaluation of endoscope geometric distortion. J. Biomed. Opt..

[23] Sun C., Guo X., Wang P., Zhang B. (2017). Computational optical distortion correction based on local polynomial by inverse model. Optik.

[24] Kaufman O., Gurfil P. (2021). Spacecraft relative navigation with an omnidirectional vision sensor. Acta Astronaut..

[25] Kumar V.R., Eising C., Witt C., Yogamani S.K. (2023). Surround-view fisheye camera perception for automated driving: Overview, survey & challenges. IEEE Trans. Intell. Transp. Syst..

[26] Ma L., Chen Y.Q., Moore K.L. (2003). A new analytical radial distortion model for camera calibration. arXiv.

[27] Parente L. (2020). Development of a Low-Cost Photogrammetric Monitoring System for Timely Detection of Slope Instability. Ph.D. Dissertation.

[28] Yin W., Zang X., Wu L., Zhang X., Zhao J. (2024). A distortion correction method based on actual camera imaging principles. Sensors.

[29] Lv G., Li Z., Liu J., Feng Z. (2025). Camera radial distortion self-correction based on straight line characteristics of edges. Proceedings of the 2025 2nd International Conference on Intelligent Perception and Pattern Recognition (IPPR), Shanghai, China, 5–8 August 2025.

[30] Fan B., Dai Y., Zhang Z., He M. (2021). Fast and robust differential relative pose estimation with radial distortion. IEEE Signal Process. Lett..

[31] Kang J.C., Kim C.S., Pak I.J., Son J.R., Kim C.S. (2021). A new phase to height model in fringe projection profilometry by considering radial distortion of camera lens. Optik.

[32] Drap P., Lefevre J. (2016). An exact formula for calculating inverse radial lens distortions. Sensors.

[33] Ernould C., Beausir B., Fundenberger J.-J., Taupin V., Bouzy E. (2021). Integrated correction of optical distortions for global HR-EBSD techniques. Ultramicroscopy.

[34] Lee J.-K., Jung W.-C. (2019). Comparison between two coordinate transformation-based orientation alignment methods. J. Sens. Sci. Technol..

[35] Jaroš M. (2021). Calibration of Multiple Cameras for Autonomous Driving. Bachelor’s Thesis.

[36] Buquet J., Zhang J., Roulet P., Thibault S., Lalonde J.-F. Evaluating the impact of wide-angle lens distortion on learning-based depth estimation. Proceedings of the IEEE/CVF Conference on Computer Vision and Pattern Recognition (CVPR) Workshops.

[37] Nocerino E., Dubbini M., Menna F., Remondino F., Gattelli M., Covi D. (2017). Geometric calibration and radiometric correction of the maia multispectral camera. Int. Arch. Photogramm. Remote Sens. Spat. Inf. Sci..

[38] Shu S., Fu Y., Liu S., Zhang Y., Zhang T., Wu T., Gao X. (2024). A correction method for radial distortion and nonlinear response of infrared cameras. Rev. Sci. Instrum..

[39] Stansberry D. (2023). Calibration of In-Situ Shape Sensing Spar. Master’s Thesis.

[40] Zhou P., Gu C., Zhang W., Yang Z., Zhang Y., Cai W., Zhou G. (2020). Light field endoscope calibration based on virtual objective lens and virtual feature points. Opt. Eng..

[41] Peng T., Gu A., Fang D., Zhang Z. (2025). High-precision camera calibration based on concentric circle compensation for industrial environments. Opt. Express.

